# Occupational risk factors for suicide: A systematic review and meta-analysis

**DOI:** 10.1097/MD.0000000000049903

**Published:** 2026-08-07

**Authors:** Sohrab Amiri, Moien A.B. Khan

**Affiliations:** a Spiritual Health Research Center, Lifestyle Institute, Baqiyatallah University of Medical Sciences, Tehran, Iran; b Department of Family Medicine, College of Medicine and Health Sciences, United Arab Emirates University, Al Ain, United Arab Emirates.

**Keywords:** job, meta-analysis, occupational risk factor, suicidal ideation, suicide, suicide attempt, systematic review

## Abstract

**Background:**

Suicide is one of the major health problems that imposes a heavy burden on the health system. Currently, there is a notable absence of review and meta-analysis studies capable of exploring a broader spectrum of occupational factors associated with dimensions of suicide. This study was conducted to investigate occupational risk factors related to suicide dimensions by systematic review and meta-analysis.

**Methods:**

Preferred Reporting Items for Systematic Reviews and Meta-analyses have been used. The databases searched in this study included: PubMed, Web of Science, and Scopus. The defined period included the beginning of the formation of these databases until August 2023, and this search was limited to studies published and available in English. The random effects method was used in a pool of different studies. In this research, the odds ratio with a 95% confidence interval (CI) was used in the report of findings.

**Results:**

The relationship between job demands and suicide dimensions was equal to the odds ratio of 1.29 with a CI between 1.11 and 1.50. This relationship was equal to the odds ratio1.15 with a CI between 1.08 and 1.23. Also, other risk factors for suicide were: conflict at work, job strain, job insecurity, workplace bullying, job stress, burnout, and work-family conflict.

**Conclusion:**

This study showed how much the work environment and related risk factors can play a role in different aspects of suicide. Therefore, in the policies related to health at work, it is necessary to pay attention to the field of monitoring and screening of mental health in the work environment.

## 1. Introduction

Suicide is one of the health-related phenomena that imposes a heavy burden on society^[1]^; so this phenomenon is recognized by the World Health Organization (WHO) as an important public health issue.^[2]^ Suicide is defined as an intentional death caused by self-directed injury^[3]^, whose rate is different based on gender and age group.^[4,5]^ According to the latest report published by WHO in 2023, more than 700,000 people in the world die by suicide every year.^[6]^ More than 77% of suicides occurred in low- and middle-income countries in 2019.^[6]^ There are far more people with suicide attempts than there are deaths by suicide.^[6]^ A suicide attempt is defined as “non-fatal, self-directed, potentially injurious behavior with intent to die as a result of the behavior. A suicide attempt might not result in injury and suicidal ideation is defined as “thinking about, considering, or planning suicide.”^[7]^ The lifetime prevalence of suicide attempts in the general population is reported to be 0.8%.^[8]^ The ratio of suicide in men to women is reported to be 1.7.^[9]^ In another study, the lifetime prevalence of suicidal ideation and suicide attempts was reported as 13.5% and 4.6%, respectively.^[10]^ According to the nature of the suicide phenomenon, various risk factors have been expressed in this regard, including mental illness,^[11–14]^ physical diseases,^[15–18]^ body mass index,^[19]^ low income and unemployment,^[20–22]^ immigration,^[23]^ alcohol and substance use,^[24,25]^ and eating disorders.^[26]^ One of the important factors in mental health is the work environment in which a person works.^[27]^

Psychosocial factors in which people work are known as important factors in health.^[28]^ Various studies show that facing psychosocial risks in the workplace is associated with an increased risk of mental health problems.^[27,29]^ People who experience high job strain are at risk of depression, so this group is 77% more exposed to clinical depression,^[30]^ it is also associated with increased burnout,^[31]^ musculoskeletal pain,^[32]^ and mortality.^[33]^ Workplace bullying is associated with an increased risk of sleep problems,^[34]^ sickness absence,^[35]^ depression,^[36,37]^ and anxiety.^[38]^ The existing literature about the role of occupational risk factors and mental health is very extensive. Other factors that can play a role in different aspects of suicide are occupational risk factors.^[39,40]^

Studies on occupational risk factors for suicide have yielded some results, according to which workplace bullying has been associated with the odd risk of suicidal ideation and behavior 2.03-times and 2.67-times, respectively.^[39]^ Also, another meta-analysis study on occupational stressors and suicidality has shown that poor social support at work, job insecurity, and low job control are associated with suicidal ideation or suicidal mortality.^[40]^ Also, some other studies are limited to the role of working conditions,^[41]^ job strain,^[42]^ and workplace^[43]^ bullying with suicidal ideation and behavior. Another study has demonstrated that stress and job-related factors significantly influence cognitive functions, leading to notable impairments in areas such as verbal memory, planning, cognitive flexibility, set-shifting, and both simple and complex attention among middle-aged miners when compared to control groups.^[44]^ The findings also highlight that the duration of employment and the level of education play a crucial role in shaping executive functions.^[44]^

Although the studies conducted on occupational factors for mental health are very extensive, and the occupational factors related to the dimensions of suicide are very extensive, there are no review and meta-analysis studies that can examine a wider range of occupational factors related to suicide dimensions and provide a comprehensive review. The review and meta-analysis studies mentioned in the previous sections have only addressed some occupational risk factors or only some aspects of suicide, such as suicidal thoughts and suicide attempts, which have been examined.

While there are various review studies and meta-analyses that examine the connection between occupational factors and instances of suicide ^[40–43]^, these works often fall short of offering a truly comprehensive perspective. Within this domain of prior research, suicide has garnered relatively limited attention despite representing the most extreme manifestation of suicidal behavior. Notably, certain studies have exclusively focused on male subjects ^[45]^, neglecting to account for both sexes in their analyses. Research on suicidal ideation has been less frequently subjected to meta-analysis when examining its connection to occupational risk factors.^[46]^ Occupational risk factors like workplace conflict, job-related stress, and burnout have not been thoroughly assessed in relation to their potential impact on different forms of suicidal behavior, such as suicidal thoughts, attempts, or suicide mortality.

The references mentioned in earlier discussions make it evident that certain critical aspects of occupational health, particularly those tied to suicidal behaviors, remain underrepresented or entirely absent in prior research. This gap highlights the need for a more exhaustive exploration of how workplace-related elements contribute to such behaviors, ensuring that overlooked dimensions are brought to light for a fuller understanding. This study offers a more up-to-date and thorough exploration of the evidence concerning the connection between various occupational factors and the risk of suicide. By delving deeper into the intricacies of this relationship, the research aims to shed light on how job-related variables might influence mental health outcomes, ultimately contributing to a broader understanding of potential risk factors linked to suicide within workplace settings. Based on what was stated, the purpose of this research was to conduct a systematic review and meta-analysis to investigate occupational risk factors related to suicidal ideation, suicide attempts, and suicide mortality.

## 2. Method

### 2.1. Protocol

In the current research, the steps recommended in Preferred Reporting Items for Systematic Reviews and Meta-analyses have been used to conduct this research.^[47]^

### 2.2. Inclusion and exclusion criteria

The criteria for inclusion in this study were as follows: First, studies employing cross-sectional, longitudinal, or case-control designs were deemed eligible for consideration. Second, to qualify, these studies needed to report on the association between occupational risk factors and at least one aspect of suicide, such as suicidal ideation, suicide attempts, or suicide mortality. Finally, the metrics used to evaluate the relationship between occupational risk factors and suicide had to include one of the following: odds ratio, risk ratio, or relative risk with a 95% confidence interval (CI), or correlation coefficients accompanied by sample size.

The following categories of studies were deemed ineligible for inclusion in this research: studies employing case study or review-based methodologies, and investigations examining the relationship between a specific occupational risk factor and suicide, where meta-analysis or data pooling with other studies was unfeasible due to an insufficient number of studies addressing that particular relationship. Pooling studies should have a minimum threshold. This means that there was a single study that could not be pooled. Studies that failed to provide the essential data required for calculating odds ratios were excluded. Additionally, in cases where multiple studies were derived from the same dataset, only 1 study was chosen for inclusion.

### 2.3. Information sources

In the search for studies for this research, 3 databases were systematically searched using a comprehensive syntax, which is mentioned in Supplemental Digital Content 1, Supplemental Digital Content 1. The databases searched in this study included: PubMed, Web of Science, and Scopus. The defined period included the beginning of the formation of these databases until August 2023, and this search was limited to studies published and available in English.

### 2.4. Search strategy

In Supplemental Digital Content 1, Supplemental Digital Content 1, the syntax of keywords is included, which is based on different dimensions of occupational risk factors as an exposure variable and suicide as an outcome variable.

### 2.5. Selection process

**Figure 1.**
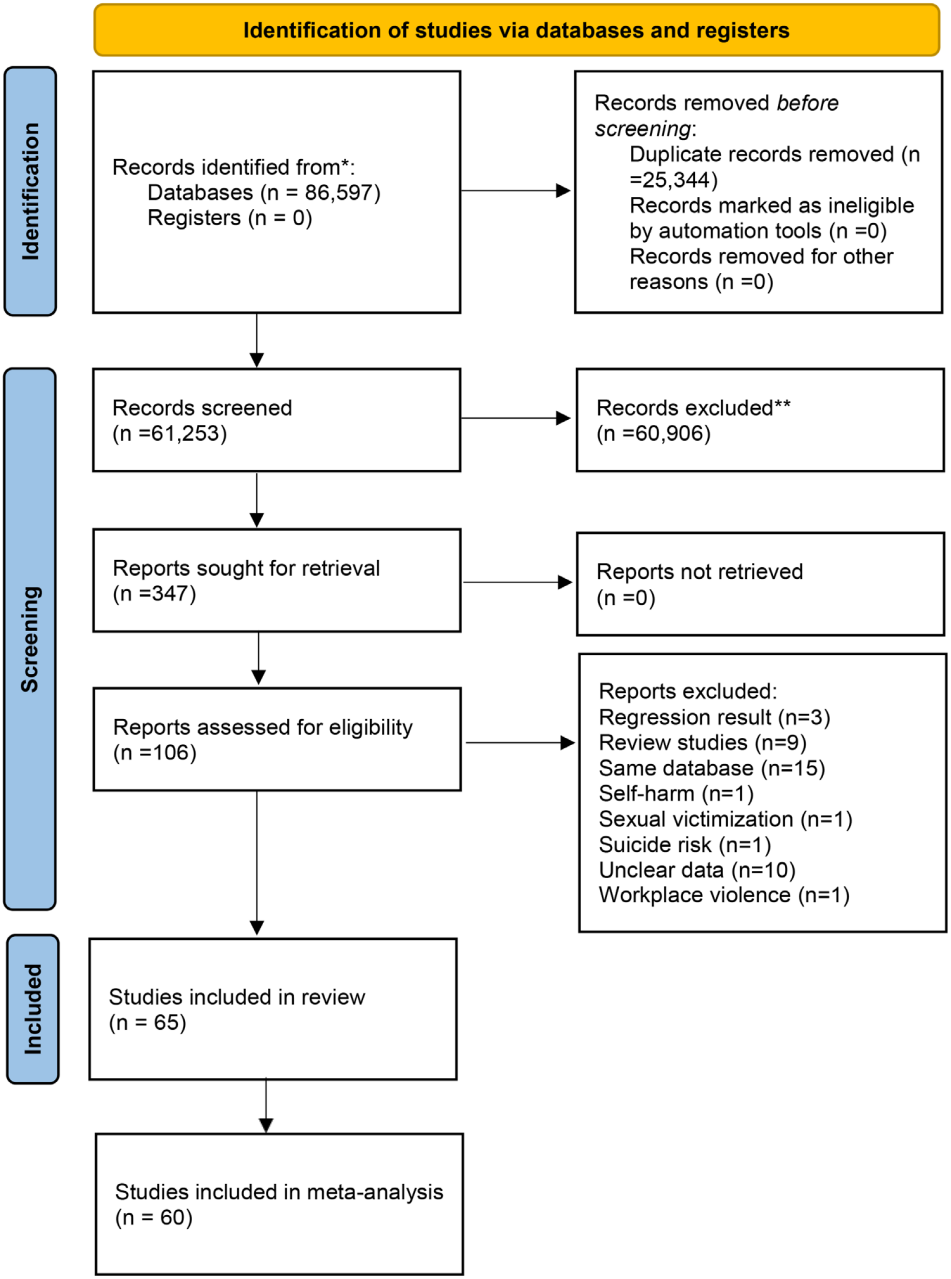
Flowchart of studies screening for this study.

The process of selecting studies is based on Figure 1, which includes 3 main steps: identification, screening, and inclusion. The studies were screened and selected based on the titles, then based on the abstract, and finally based on the full text. In the search process of selecting studies, some studies were not found, and the authors were contacted; only 4 authors responded.

### 2.6. Data collection process

In the data collection process, the researchers entered jointly the extracted data, including the following items: Authors and year of publication of the study, country, type of population, demographic characteristics of participants, type of exposure variable, type of outcome variable, and its measurement method, statistical result, and controlled variables. Data extraction was conducted independently by researchers.

### 2.7. Data items

Occupational risk variables that were defined as exposure variables in this research include: job demands, job control, conflict at work, job insecurity, lack of reward, occupational climate, work-family conflict, job strain, burnout, workload, job satisfaction, long working hours, job stress, workplace bullying, social support at work, and shift work. The outcome variables included 3 subjects related to suicide: suicidal ideation, suicide attempts, and suicide mortality.

### 2.8. Quality assessment

Effective Public Health Practice Project Quality Assessment Tool^[48,49]^ was used to measure the qualitative dimensions related to each of the studies. Based on this, 4 dimensions of this adapted tool were used in the present study: selection bias, confounders bias, data collection method, and withdrawals and dropouts.

### 2.9. Effect measures

For the pool of different studies in the meta-analysis, the odds ratio and 95% CI were used as the reporting method.

### 2.10. Statistical analysis

After extracting data from each study, these data were pooled based on the levels of independent and dependent variables. In some studies, multiple levels of a variable were reported, and these were pooled using the fixed effects method. The random effects method was used in a pool of different studies. In this research, the odds ratio with a 95% CI was used in the report of findings. For dependent exposure variables, existing procedures were used to pool them.^[50]^ This approach is based on the average of the sum of dependent outcomes. Publication bias and heterogeneity across studies were also examined using the heterogeneity chi-squared test and *I*^2^,^[51,52]^ funnel plot, Egger’s test, and the trim-and-fill method.^[53–55]^ In the review of publication bias, the number of 10 studies was considered as a cutoff point.^[56]^ Stata version 14 software (Stata Corp., College Station, TX) and Comprehensive meta-analysis-3 software^[57]^ were used for analysis.

## 3. Result

### 3.1. Study selection

Figure 1 shows the stages of selection and exclusion of studies. After screening the studies based on the inclusion and exclusion criteria, sixty-five studies were found to be eligible. Among these studies, 5 studies^[58–62]^ that reported the hazard ratio were excluded, and finally, sixty studies^[63–122]^ were included in the meta-analysis, and the detailed characteristics of each study are listed in Table S1, Supplemental Digital Content 2.

### 3.2. Study characteristics

From the total number of studies included in this research, which included sixty-six studies, 3 were selected from cross-sectional, longitudinal, and case-control designs. These included genders and age groups, mostly adults. For each of these studies, a set of data is reported in Table S1, Supplemental Digital Content 2.

### 3.3. Risk of bias in studies

The 4 dimensions of measuring the quality of the studies (selection bias, confounder bias, data collection method, and withdrawals and dropouts) are included in Table S1, Supplemental Digital Content 2 for each of the studies. What is reported indicates the level of low, medium, and high quality of the different studies?

### 3.4. Results of individual studies

Various indices were reported for the relationship between exposure and outcome variables, the most common of which was the odds ratio with a 95% CI, and this ratio was used to report the pool of studies.

### 3.5. Results of analysis

**Figure 2.**
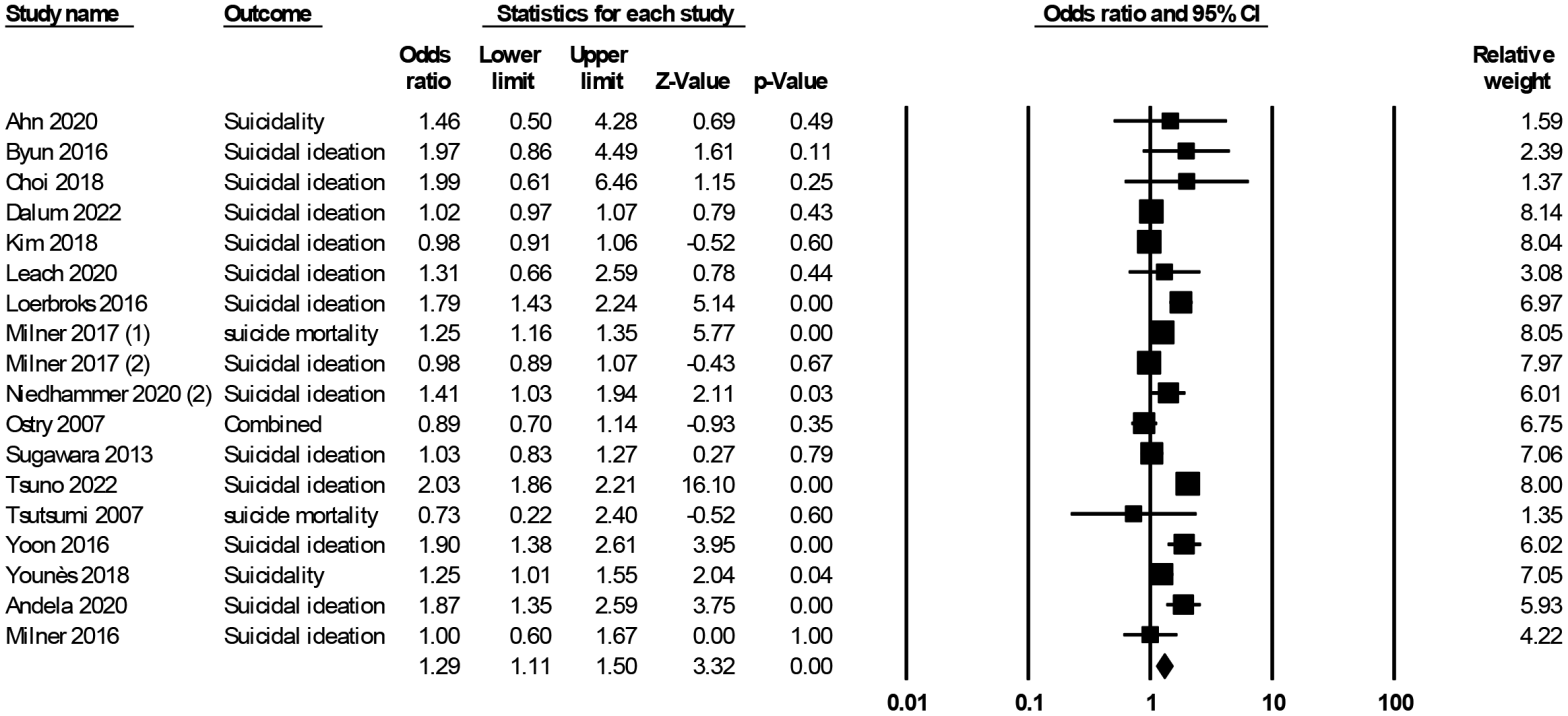
Forest plot of job demands and suicide dimensions. CI = confidence interval.

Figure 2 shows the association between job demands and suicide dimensions. This association is equal to the odds ratio1.29 with a CI between 1.11 to 1.50 (n = 18; Z = 3.31; *P* = .001; *I*^2^ = 93.63%).

**Figure 3.**
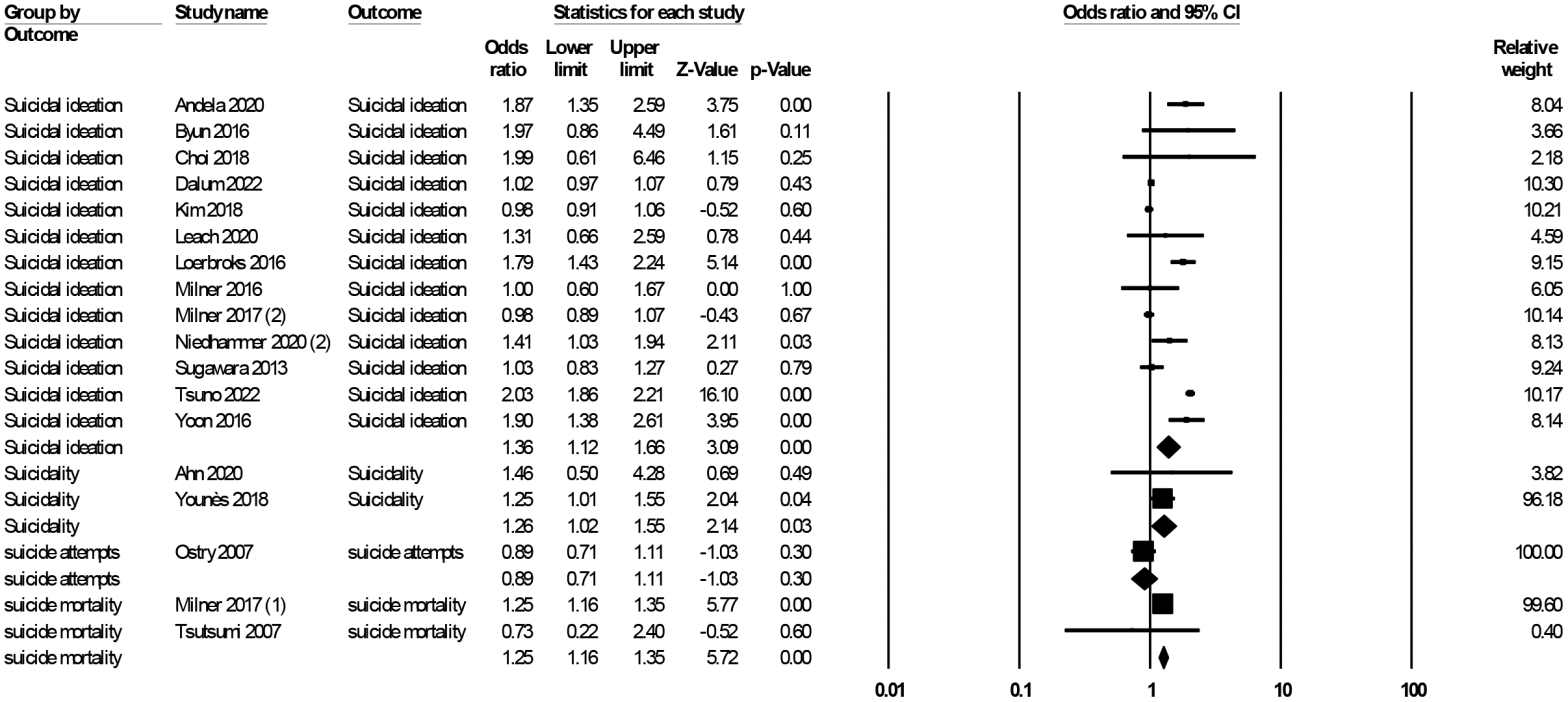
Forest plot of job demands and suicidal ideation, suicide mortality, and suicidality. CI = confidence interval.

Figure 3 shows the association between job demands and suicidal ideation, suicide mortality, and suicidality. This association is equal to the odds ratio1.36 with a CI between 1.12 and 1.66 (n = 13; Z = 3.09; *P* = .002; *I*^2^ = 95.33%) for suicidal ideation. This association is equal to the odds ratio1.25 with a CI between 1.16 and 1.35 (n = 2; Z = 5.72; *P* < .001; *I*^2^ = 0%) for suicidal mortality. This association is equal to the odds ratio1.26 with CI between 1.02 to 1.55 (n = 2; Z = 2.14; *P* = .033; *I*^2^ = 0%) for suicidality.

**Figure 4.**
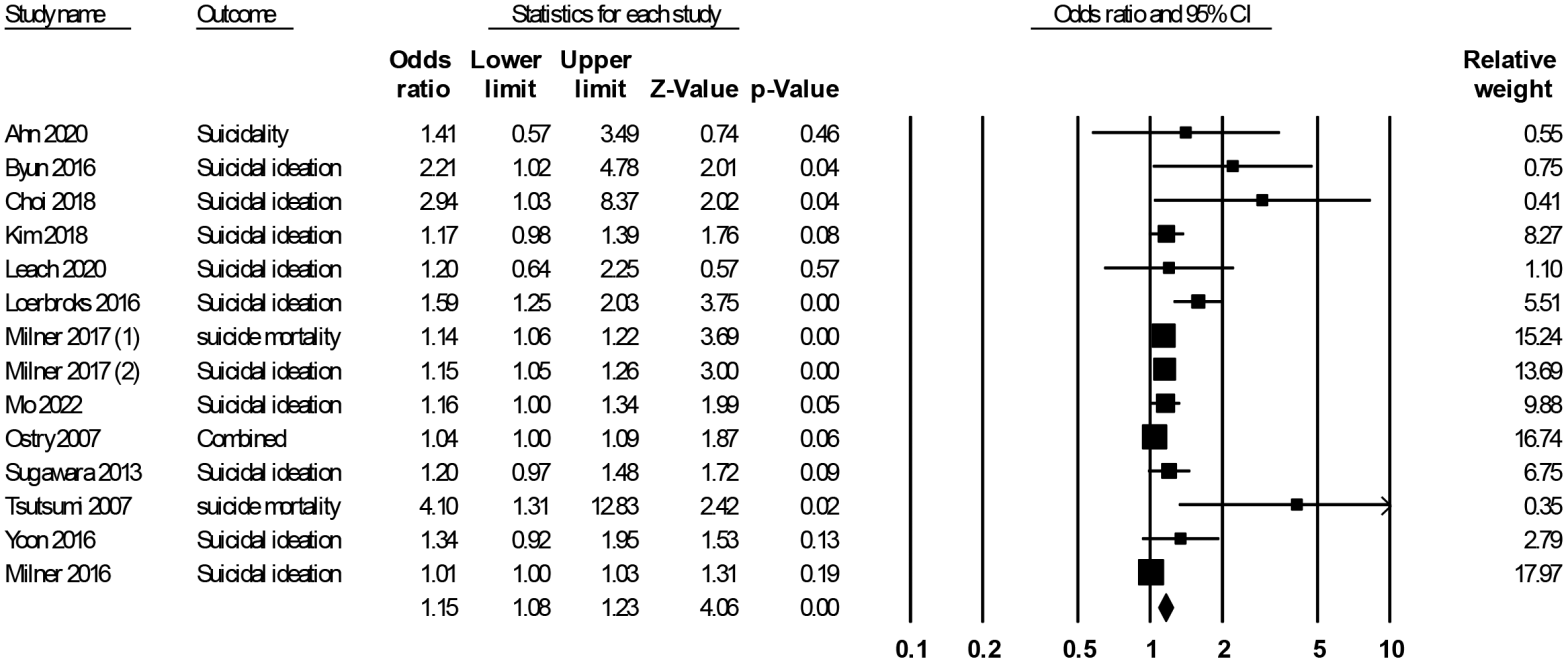
Forest plot of job control and suicide dimensions. CI = confidence interval.

Figure 4 shows the association between job control and suicide dimensions. This association is equal to the odds ratio1.15 with CI between 1.08 to 1.23 (n = 14; Z = 4.06; *P* < .001; *I*^2^ = 76.48%).

**Figure 5.**
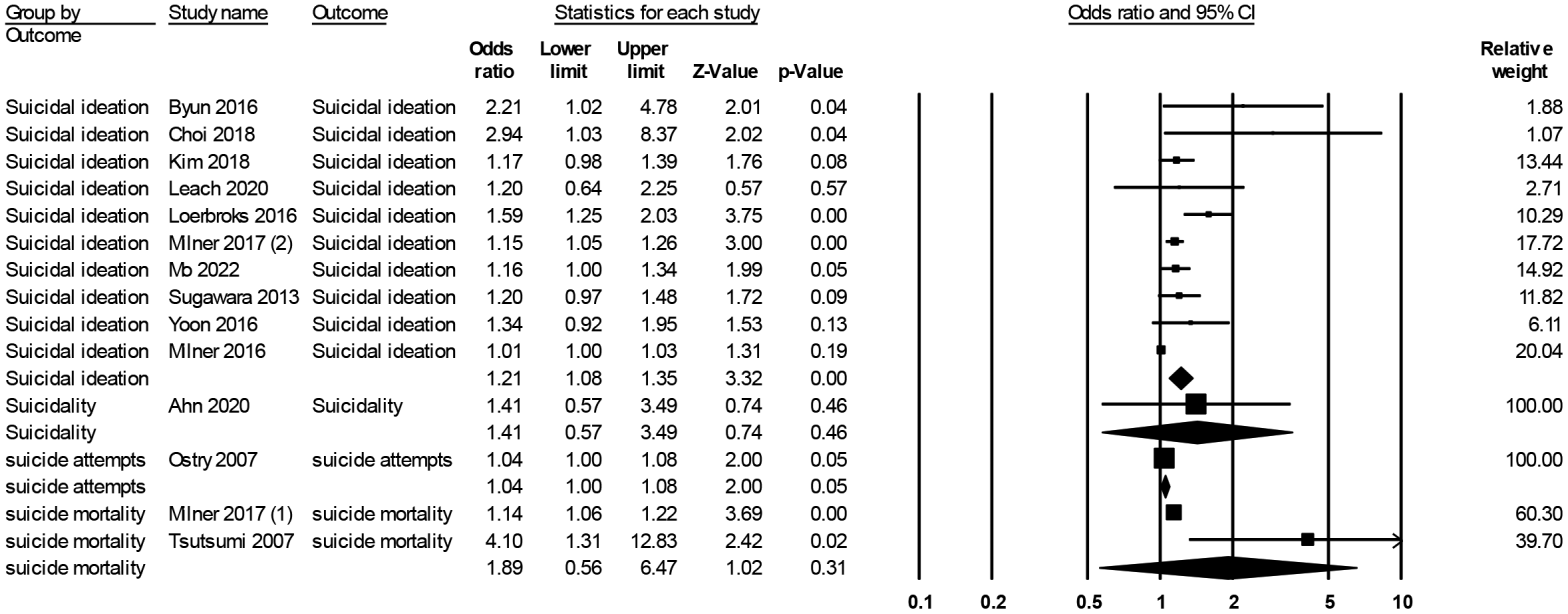
Forest plot of job control and suicidal ideation and suicide mortality. CI = confidence interval.

Figure 5 shows the association between job control, suicidal ideation, and suicide mortality. This association is equal to the odds ratio1.21 with a CI between 1.08 and 1.35 (n = 10; Z = 3.32; *P* = .001; *I*^2^ = 76.89%) for suicidal ideation. This association is equal to the odds ratio1.89 with a CI between 0.56 and 6.47 (n = 2; Z = 1.02; *P* = .307; *I*^2^ = 79.24%) for suicidal mortality.

**Figure 6.**
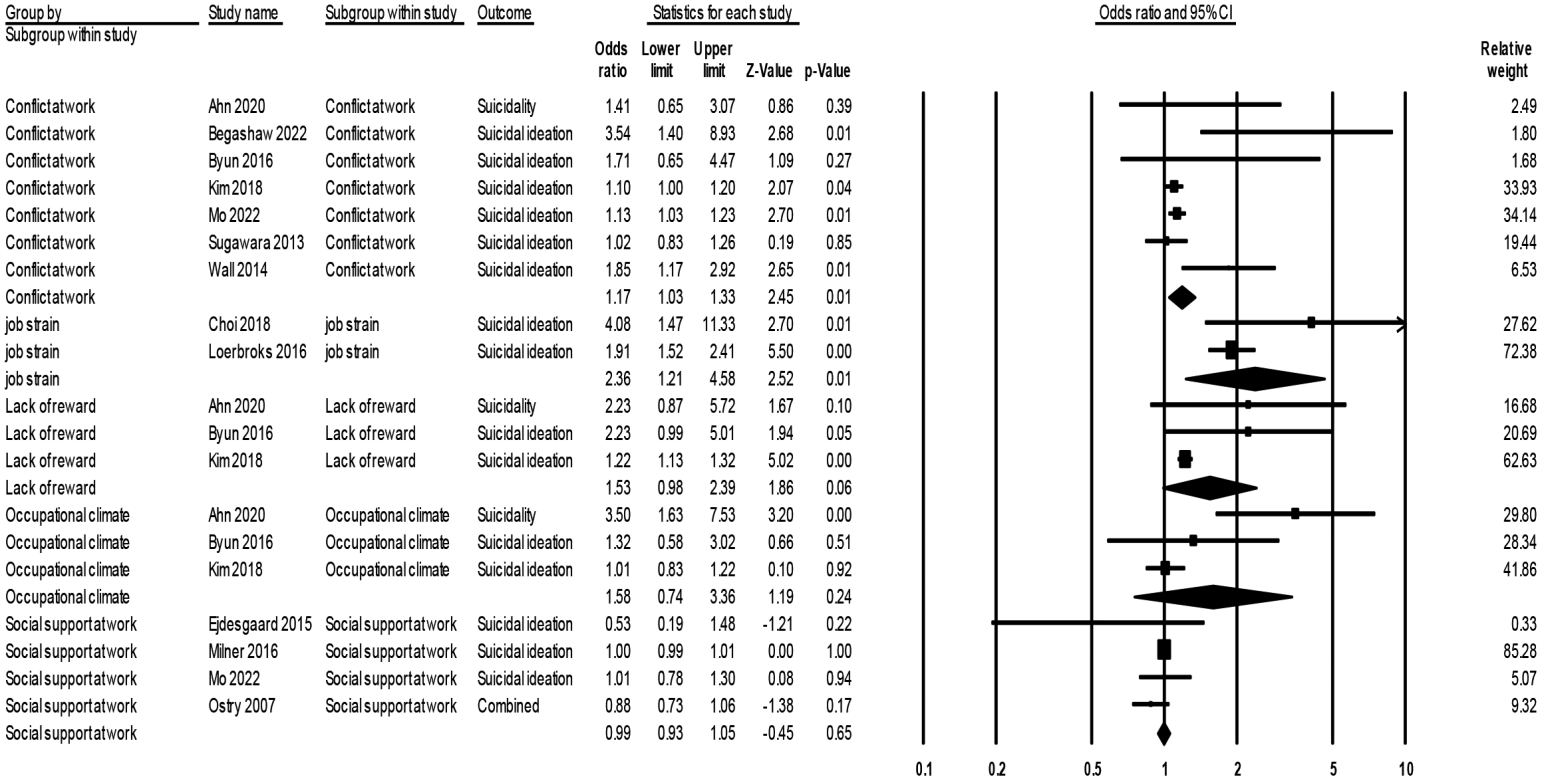
Forest plot of conflict at work, job strain, lack of reward, occupational climate, social support at work, and suicide dimensions. CI = confidence interval.

Figure 6 shows the association between conflict at work, job strain, lack of reward, occupational climate, social support at work and suicide dimensions. This association is equal to the odds ratio1.17 with a CI between 1.03 to 1.33 (n = 7; Z = 2.45; *P* = .014; *I*^2^ = 52.49%). This association is equal to the odds ratio1.17 with a CI between 1.02 and 1.34 (n = 6; Z = 2.30; *P* = .021; *I*^2^ = 59.36%) for suicidal ideation (not shown in the figure). The association between job strain and suicidal ideation is equal to the odds ratio 2.36 with a CI between 1.21 and 4.58 (n = 2; Z = 2.52; *P* = .012; *I*^2^ = 50.44%). The association between lack of reward, occupational climate, social support at work, and suicide dimensions was not significant.

**Figure 7.**
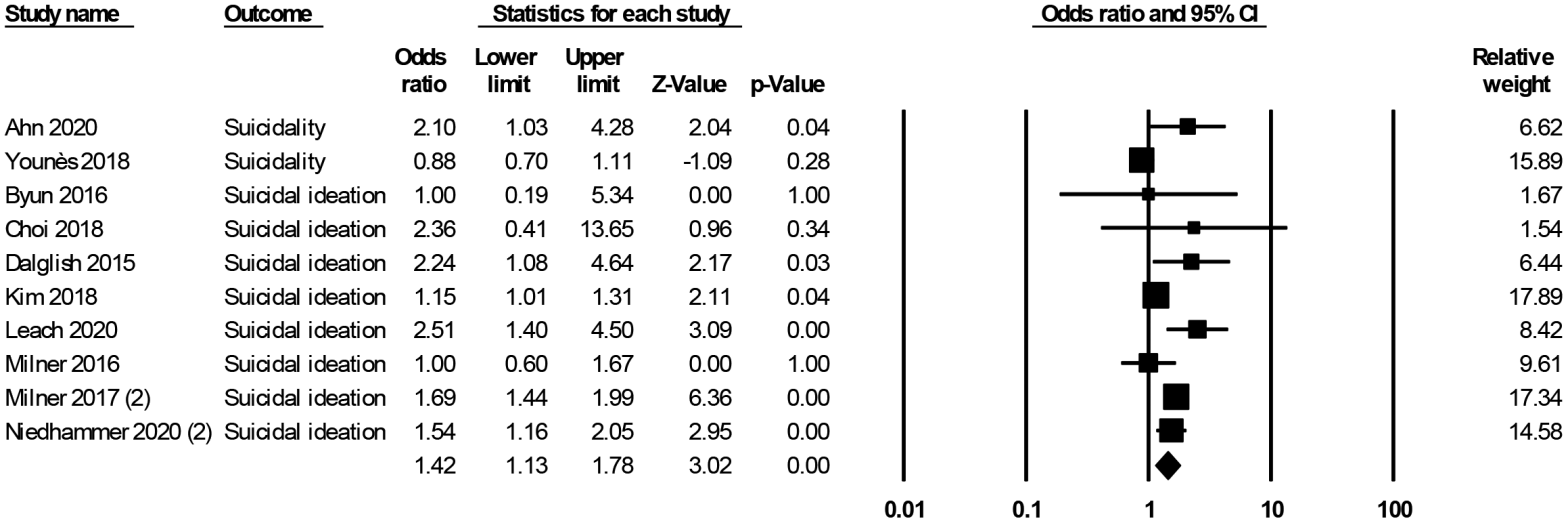
Forest plot of job insecurity and suicide dimensions. CI = confidence interval.

Figure 7 shows the association between job insecurity and suicide dimensions. This association is equal to the odds ratio1.42 with a CI between 1.13 to 1.78 (n = 10; Z = 3.02; *P* = .002; *I*^2^ = 75.11%).

**Figure 8.**
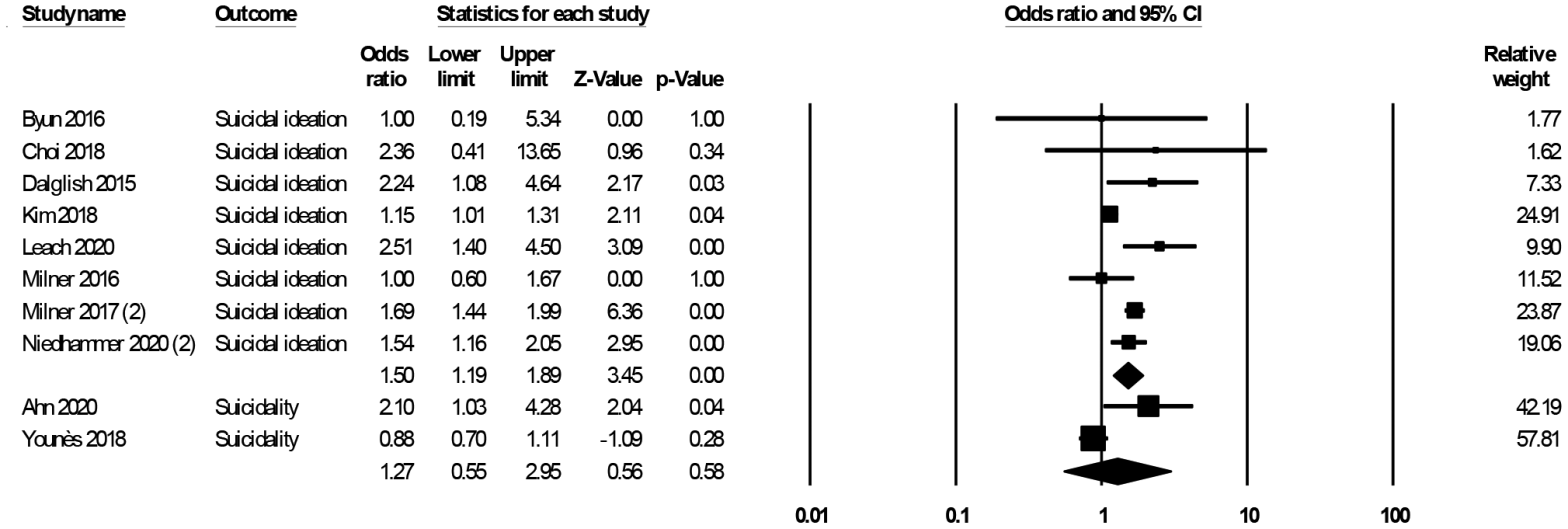
Forest plot of job insecurity, suicidal ideation, and suicidality. CI = confidence interval.

Figure 8 shows the association between job insecurity, suicidal ideation, and suicidality. This association is equal to the odds ratio1.50 with a CI between 1.19 and 1.89 (n = 8; Z = 3.45; *P* = .001; *I*^2^ = 68.01%) for suicidal ideation. This association is equal to the odds ratio1.27 with CI between 0.55 to 2.95 (n = 2; Z = 0.56; *P* = .578; *I*^2^ = 80.71%) for suicidality.

**Figure 9.**
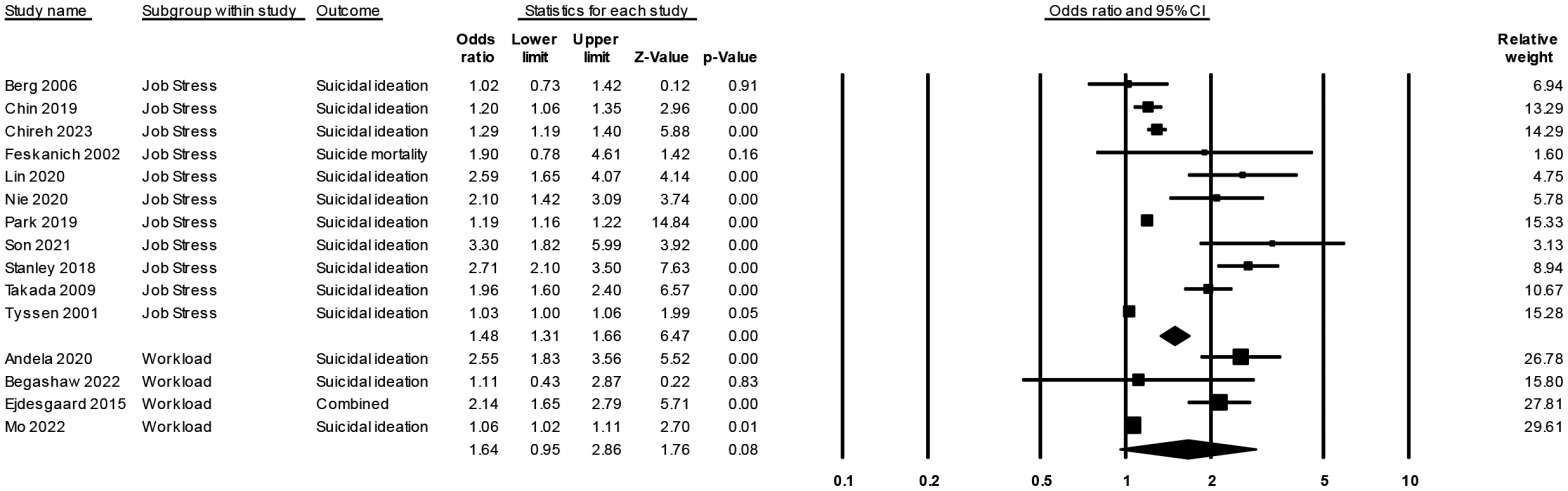
Forest plot of job stress, workload, and suicide dimensions. CI = confidence interval.

Figure 9 shows the association between job stress, workload, and suicide dimensions. This association is equal to the odds ratio1.48 with a CI between 1.31 and 1.66 (n = 11; Z = 6.47; *P* < .001; *I*^2^ = 94.34%) for job stress. This association is equal to the odds ratio1.64 with CI between 0.95 to 2.86 (n = 4; Z = 1.76; *P* = .078; *I*^2^ = 94.27%) for workload.

**Figure 10.**
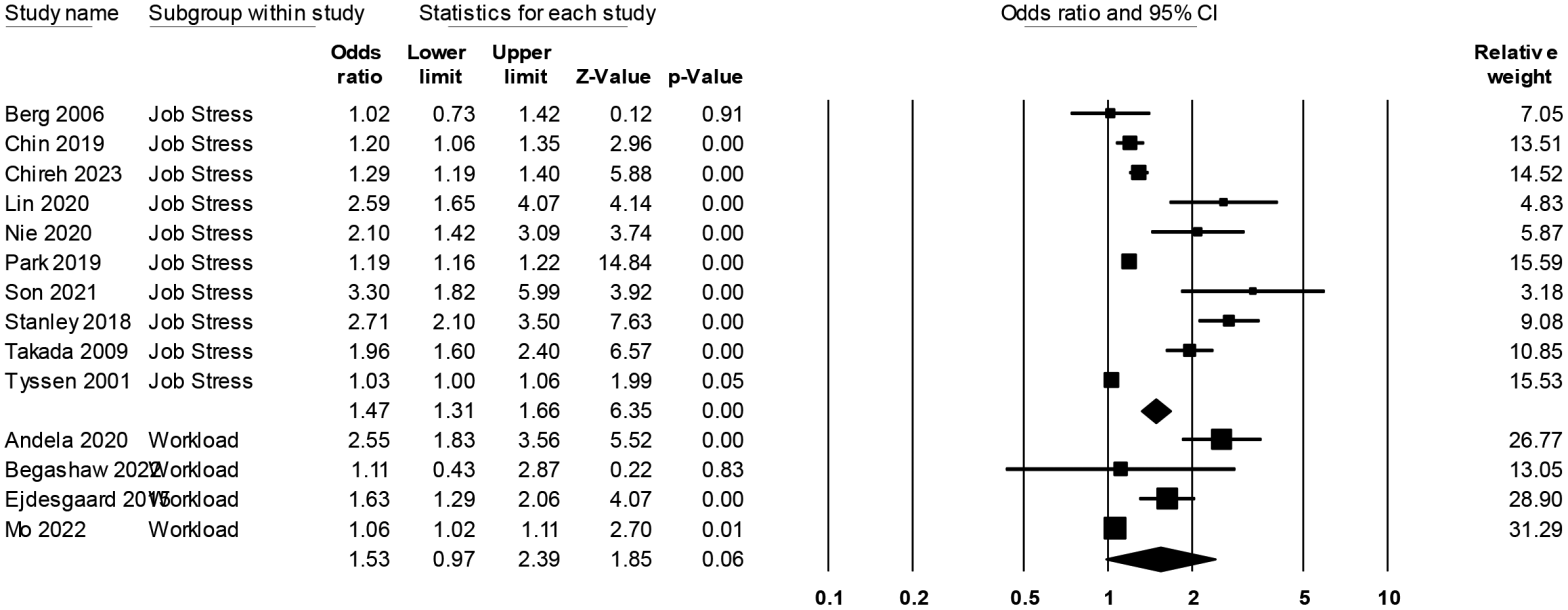
Forest plot of job stress, workload, and suicidal ideation. CI = confidence interval.

Figure 10 shows the association between job stress, workload, and suicidal ideation. This association is equal to the odds ratio1.47 with a CI between 1.31 and 1.66 (n = 10; Z = 6.35; *P* < .001; *I*^2^ = 94.87%) for job stress. This association is equal to the odds ratio1.53 with CI between 0.97 to 2.39 (n = 4; Z = 1.85; *P* = .065; *I*^2^ = 92.11%) for workload.

**Figure 11.**
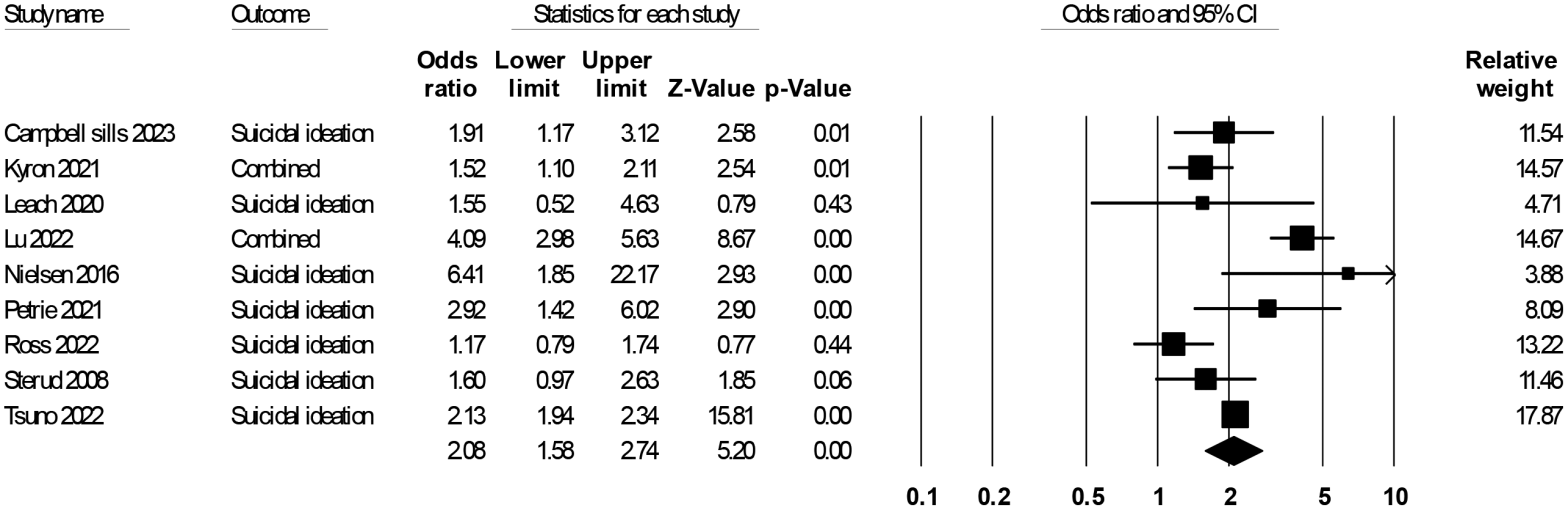
Forest plot of workplace bullying and suicide dimensions. CI = confidence interval.

Figure 11 shows the association between workplace bullying and suicide dimensions. This association is equal to the odds ratio 2.08 with a CI between 1.58 and 2.74 (n = 9; Z = 5.20; *P* < .001; *I*^2^ = 76.80%). This association is equal to the odds ratio1.96 with a CI between 1.51 and 2.54 (n = 9; Z = 5.05; *P* < .001; *I*^2^ = 85.42%) for suicidal ideation. This association is equal to the odds ratio 2.99 with a CI between 0.98 and 9.14 (n = 2; Z = 1.92; *P* = .054; *I*^2^ = 93.59%) for suicide attempts (not shown in the figure).

**Figure 12.**
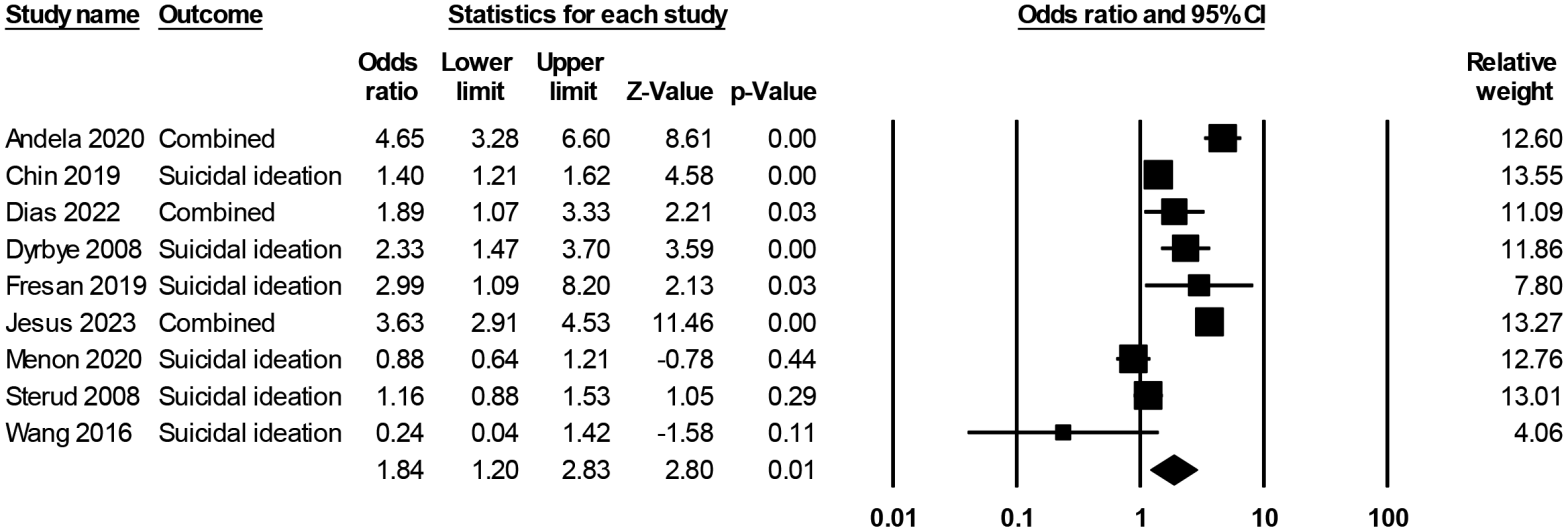
Forest plot of burnout and suicide dimensions. CI = confidence interval.

Figure 12 shows the association between burnout and suicide dimensions. This association is equal to the odds ratio 1.84 with a CI between 1.20 and 2.83 (n = 9; Z = 2.80; *P* = .005; I^2^ = 93.02%). This association is equal to the odds ratio1.73 with a CI between 1.15 and 2.60 (n = 8; Z = 2.63; *P* = .009; I^2^ = 89.75%) for suicidal ideation. (not shown in the figure).

**Figure 13.**
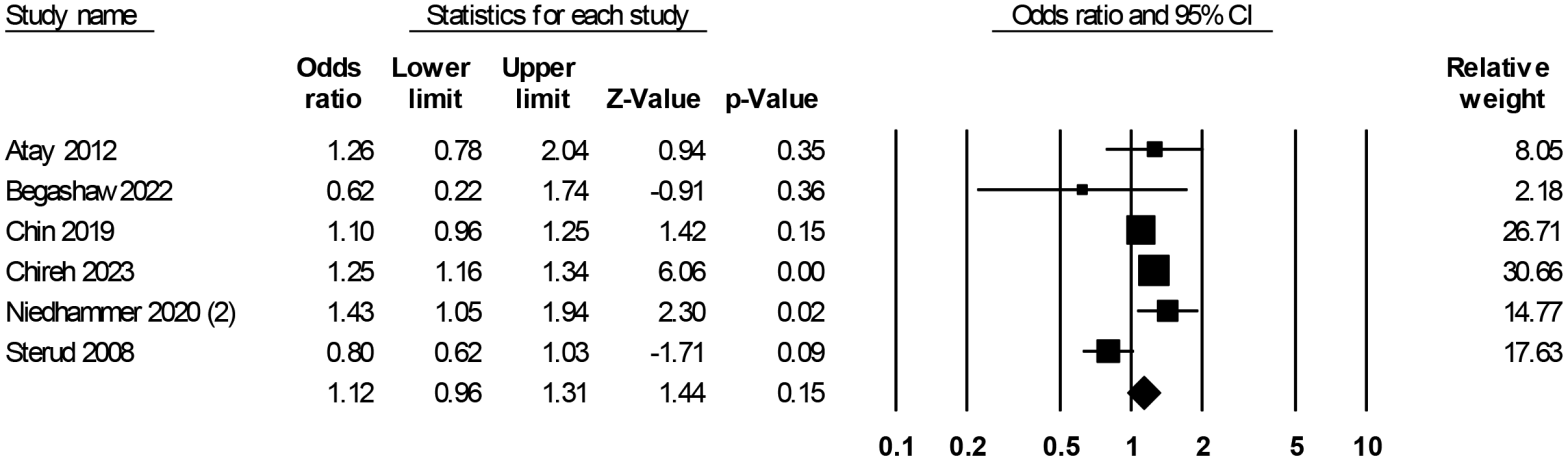
Forest plot of job dissatisfaction and suicidal ideation. CI = confidence interval.

Figure 13 shows the association between job dissatisfaction and suicidal ideation. This association is equal to the odds ratio 1.12 with a CI between 0.96 and 1.31 (n = 6; Z = 1.44; *P* = .151; *I*^2^ = 67.60%).

**Figure 14.**
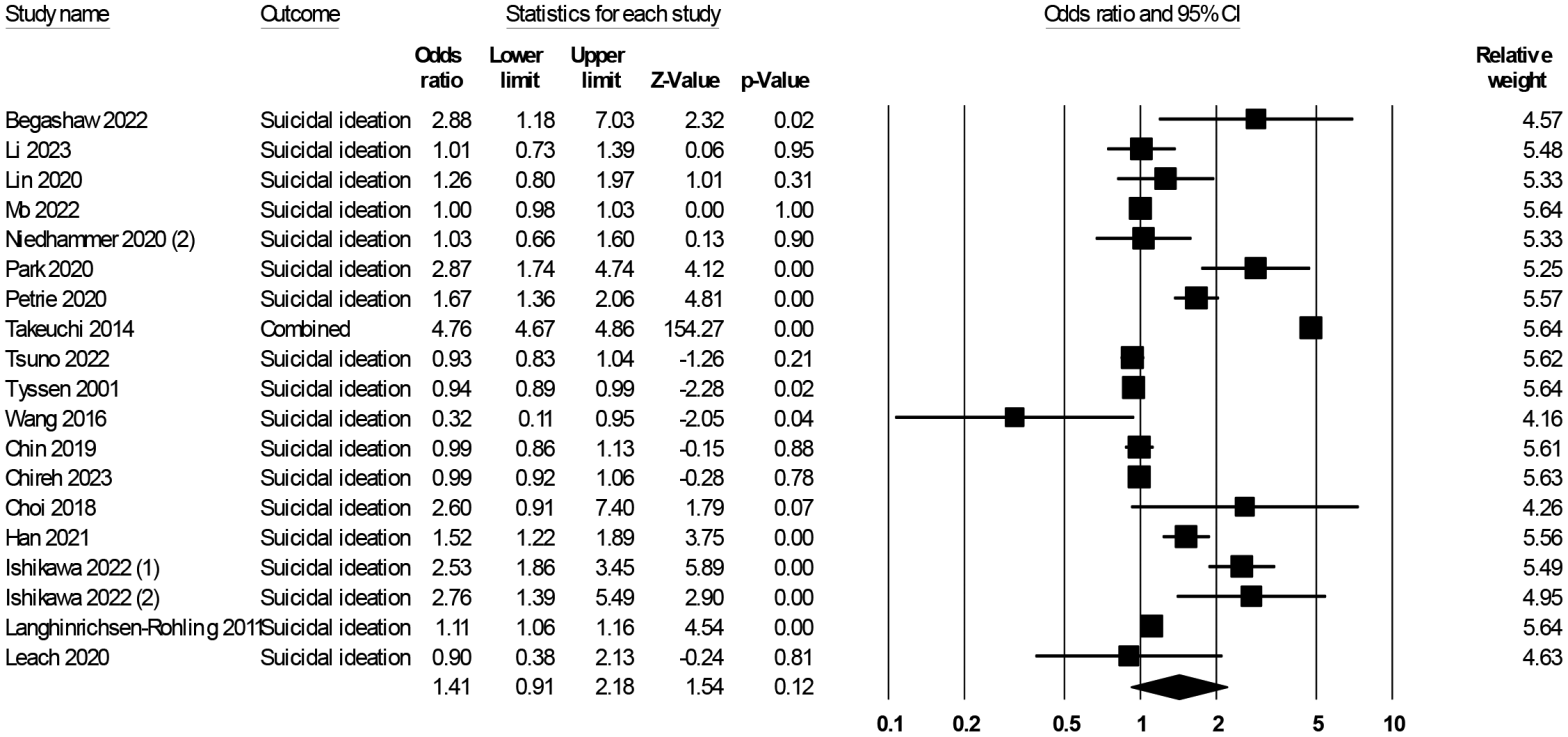
Forest plot of long working hours and suicidal ideation. CI = confidence interval.

Figure 14 shows the relationship between long working hours and suicidal ideation. This relationship is equal to the odds ratio 1.41 with a CI between 0.91 and 2.18 (n = 19; Z = 1.54; *P* = .122; *I*^2^ = 99.85%).

**Figure 15.**
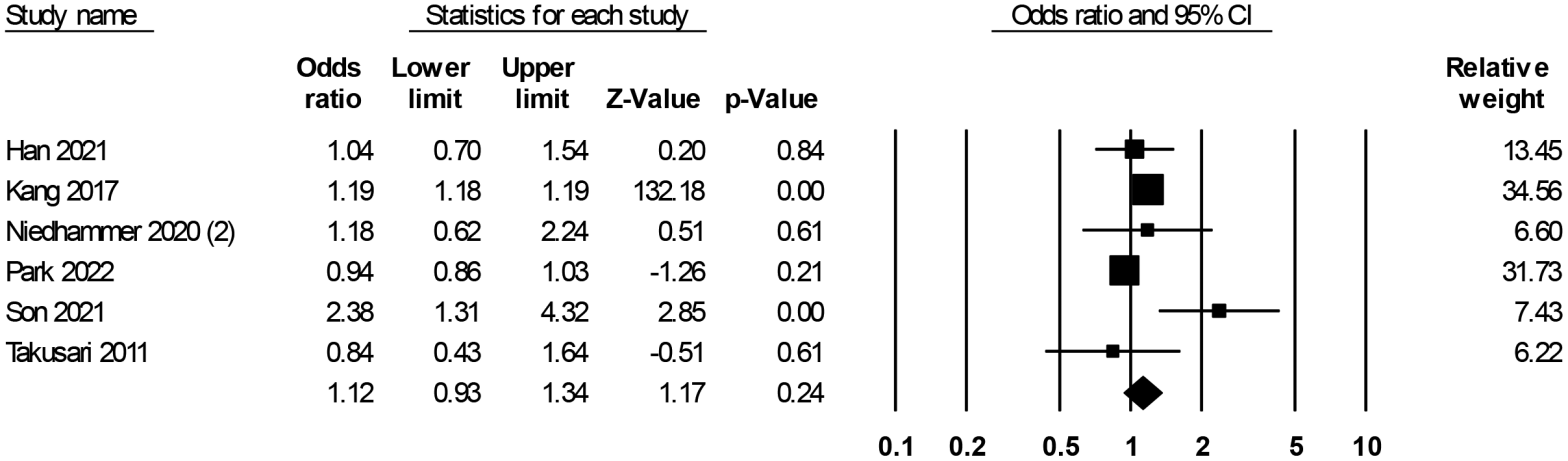
Forest plot of shift work and suicidal ideation. CI = confidence interval.

Figure 15 shows the association between shift work and suicidal ideation. This relationship is equal to the odds ratio 1.12 with a CI between 0.93 and 1.34 (n = 6; Z = 1.17; *P* = .241; *I*^2^ = 83.39%).

**Figure 16.**
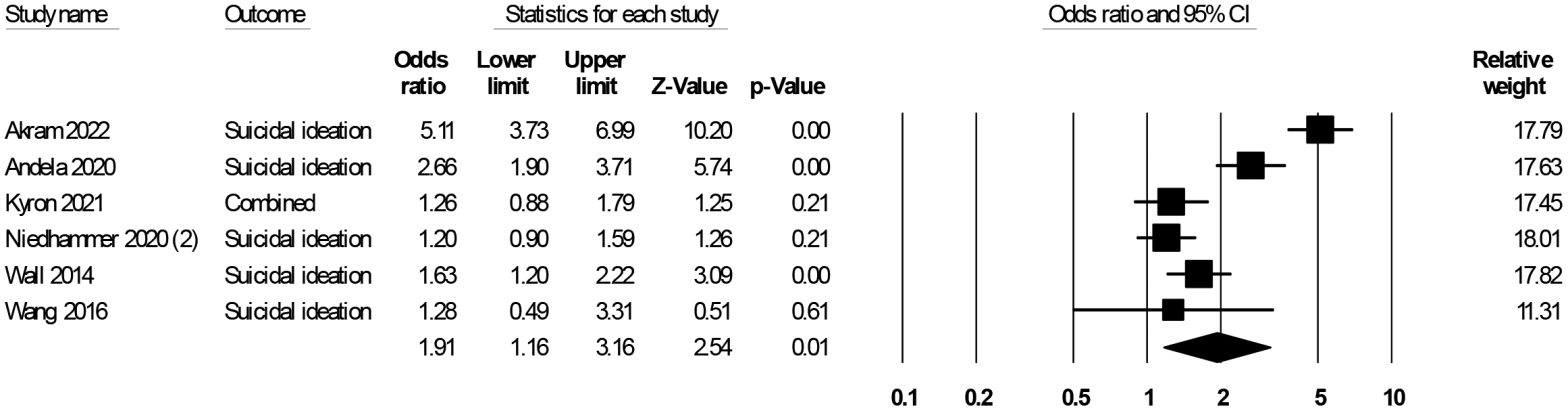
Forest plot of work-family conflict and suicide dimensions. CI = confidence interval.

Figure 16 shows the association between work-family conflict and suicide dimensions. This association is equal to the odds ratio 1.91 with a CI between 1.16 and 3.16 (n = 6; Z = 2.54; *P* = .011; *I*^2^ = 91.48%). This association is equal to the odds ratio 2.05 with a CI between 1.39 and 3.03 (n = 6; Z = 3.60; *P* < .001; *I*^2^ = 90.84%) for suicidal ideation.

### 3.6. Publication bias and heterogeneity

**Figure 17.**
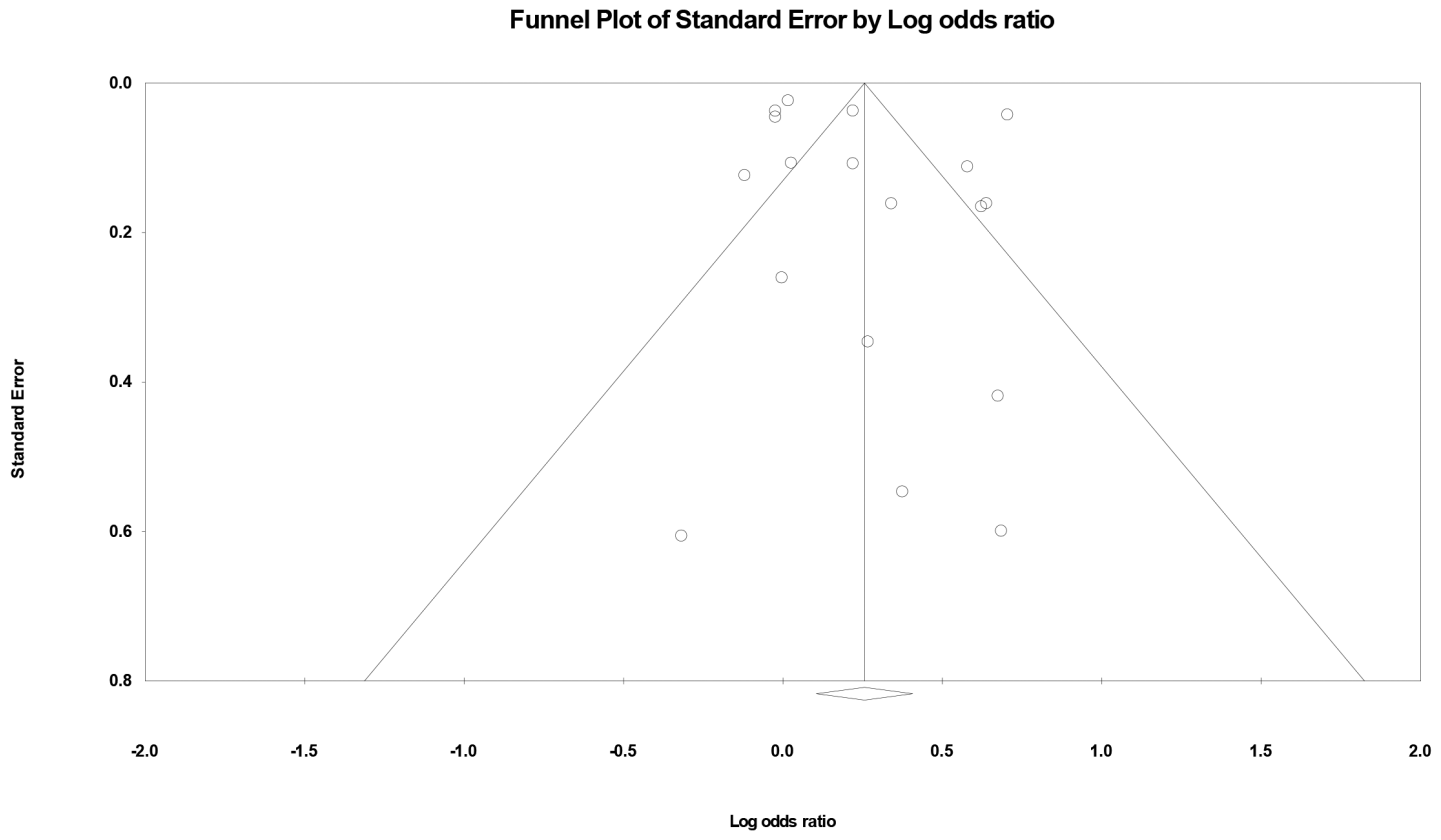
Funnel plot of job demands and suicide dimensions.

In the association between job demands and suicide dimensions, the funnel plot is in Figure 17. The Egger test was (*P* = .341), and publication bias was not shown. The trim-and-fill^[55]^ imputed 3 studies. Adjusted odds ratio was 1.21 (95% CI 1.03–1.41). Heterogeneity was *I^2^* = 93.63%, this is a high-heterogeneity^[123]^ and the heterogeneity of chi-square was 266.887 (d.f = 17; *P* < .001).

**Figure 18.**
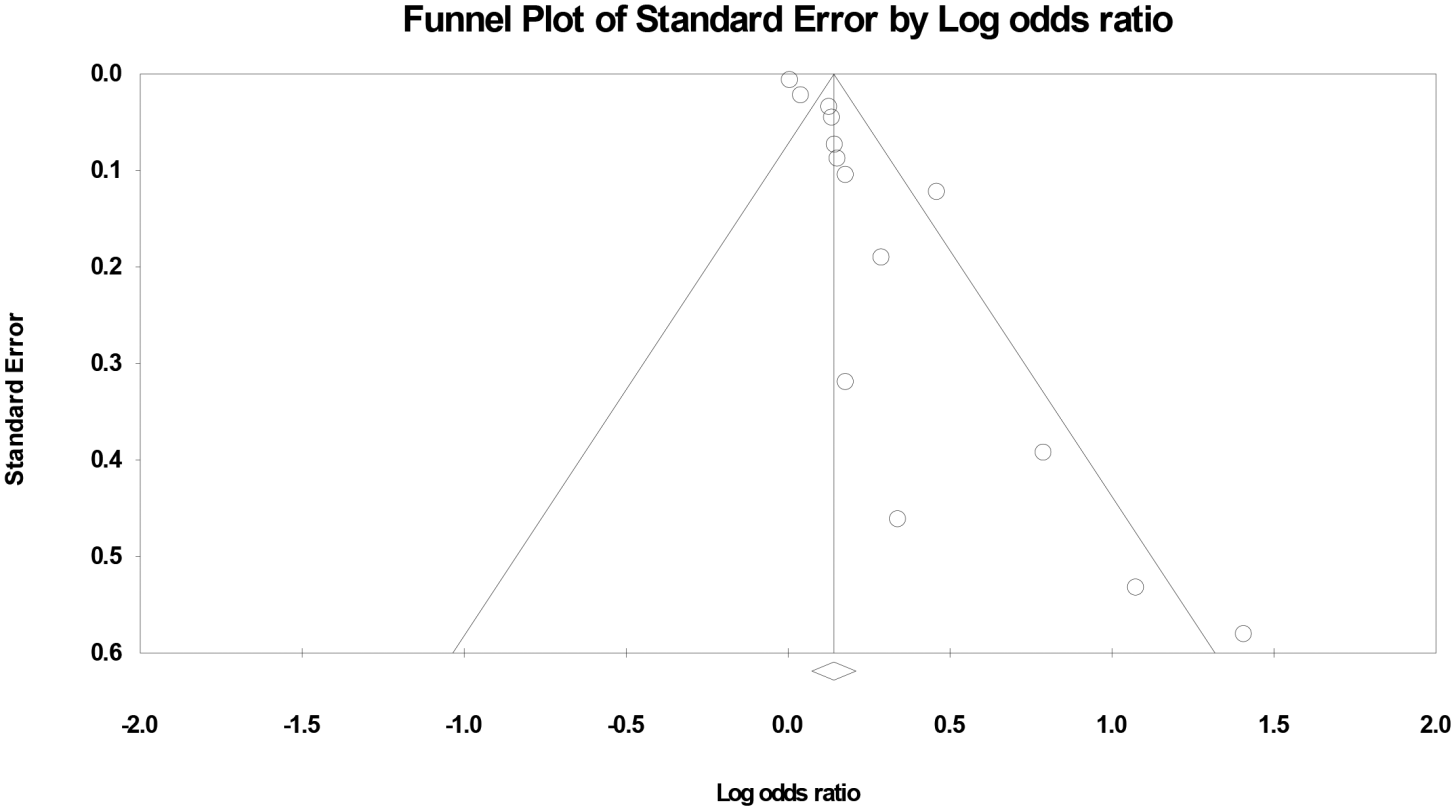
Funnel plot of job control and suicide dimensions.

In the association between job control and suicide dimensions, the funnel plot is in Figure 18. The Egger test was (*P* < .001), and publication bias was shown. The trim-and-fill^[55]^ imputed 7 studies. Adjusted odds ratio was 1.10 (95% CI 1.03–1.18). Heterogeneity was *I^2^* = 76.48%, this is a high- heterogeneity^[123]^ and the heterogeneity of chi-square was 55.262 (d.f = 13; *P* < .001).

**Figure 19.**
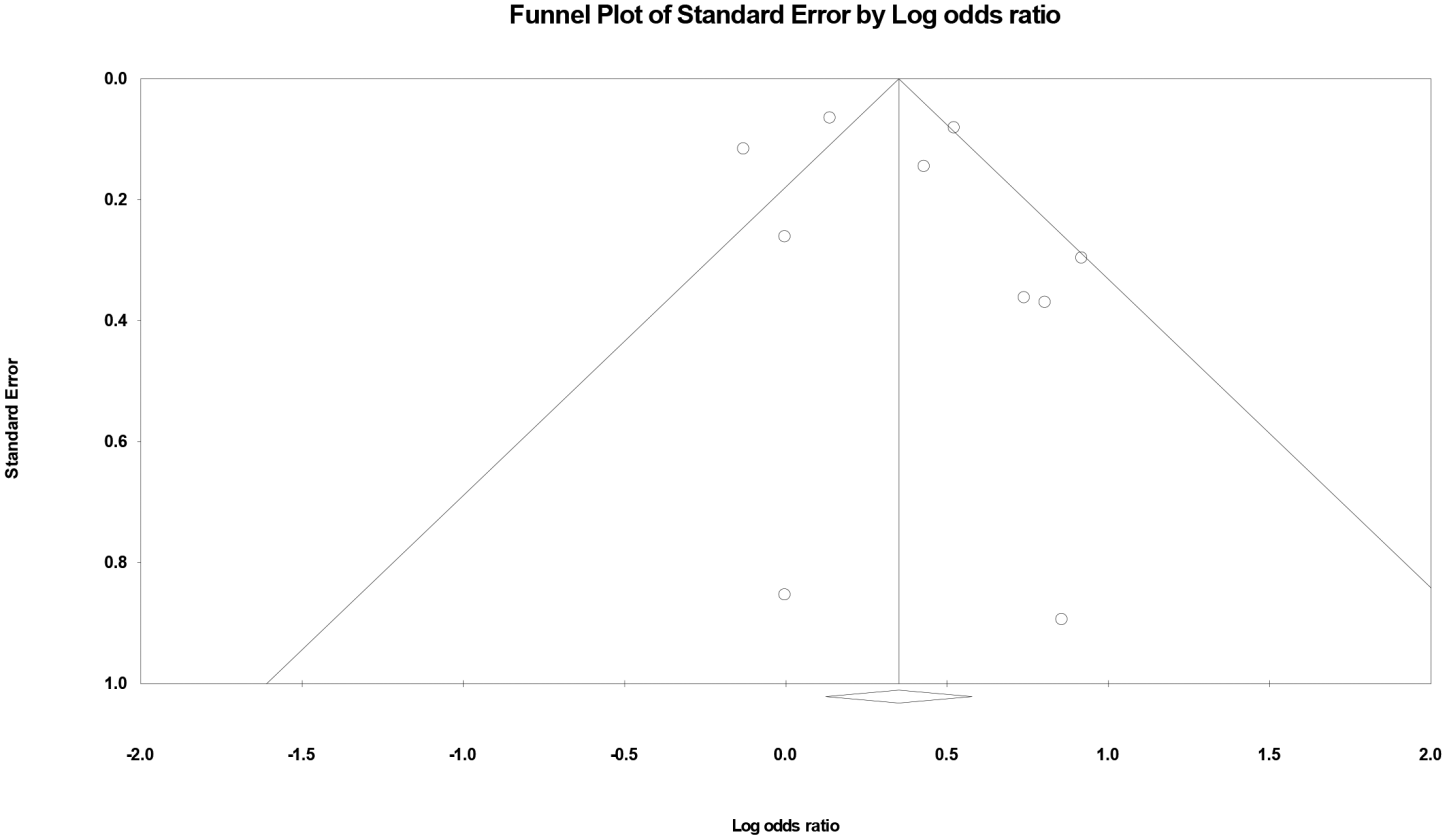
Funnel plot of job insecurity and suicide dimensions.

In the association between job insecurity and suicide dimensions, the funnel plot is in Figure 19. The Egger test was (*P* = .431), and publication bias was not shown. The trim-and-fill^[55]^ imputed 2 studies. Adjusted odds ratio was 1.33 (95% CI 1.07–1.66). Heterogeneity was *I^2^* = 75.11%, this is a high- heterogeneity^[123]^ and the heterogeneity of chi-square was 36.161 (d.f = 9; *P* < .001).

**Figure 20.**
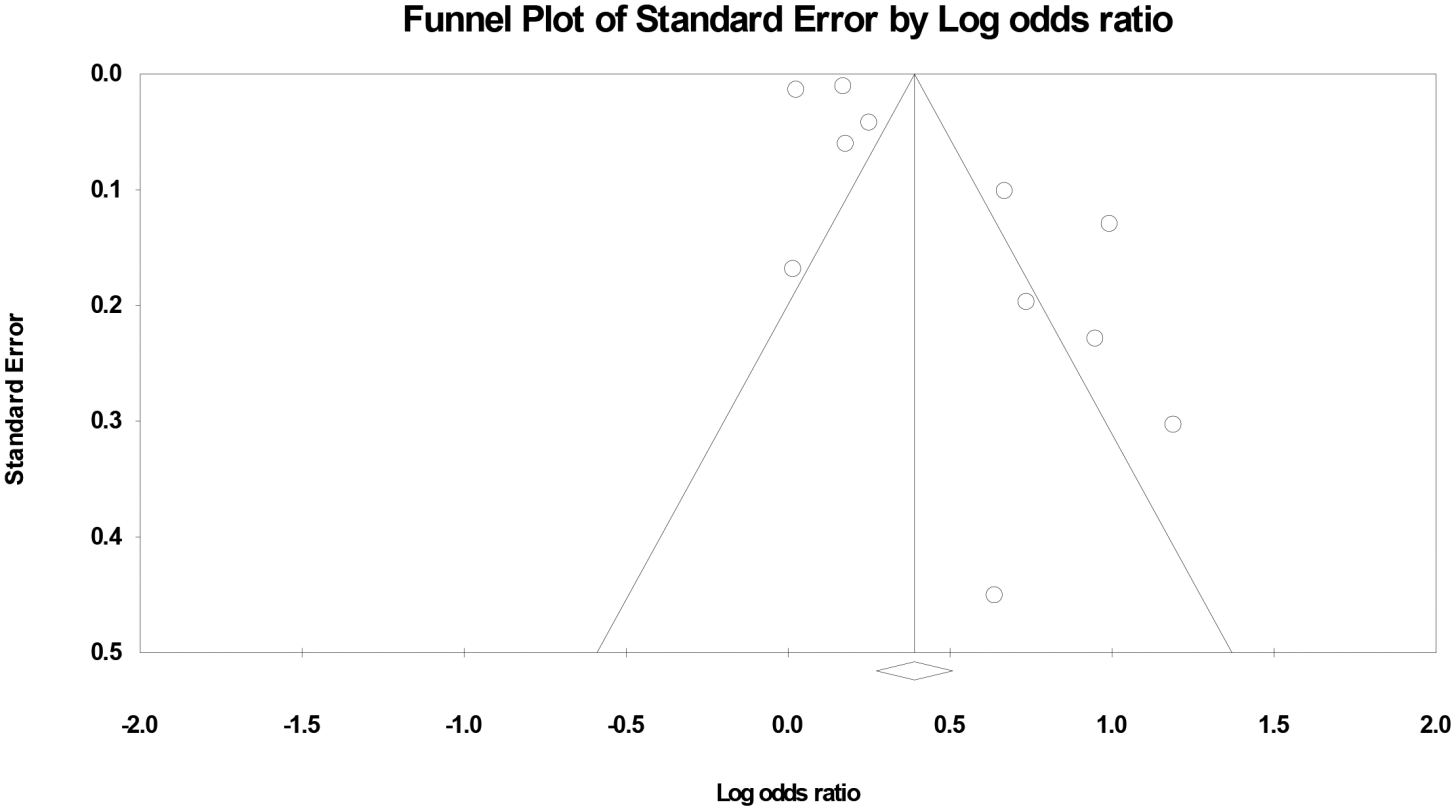
Funnel plot of job stress and suicidal ideation.

In the association between job stress and suicidal ideation, the funnel plot is in Figure 20. The Egger test was (*P* = .040), and publication bias was shown. The trim-and-fill^[55]^ imputed 4 studies. Adjusted odds ratio was 1.25 (95% CI 1.11–1.40). Heterogeneity was *I^2^* = 94.34%, this is a high- heterogeneity^[123]^ and the heterogeneity of chi-square was 176.80 (d.f = 10; *P* < .001).

**Figure 21.**
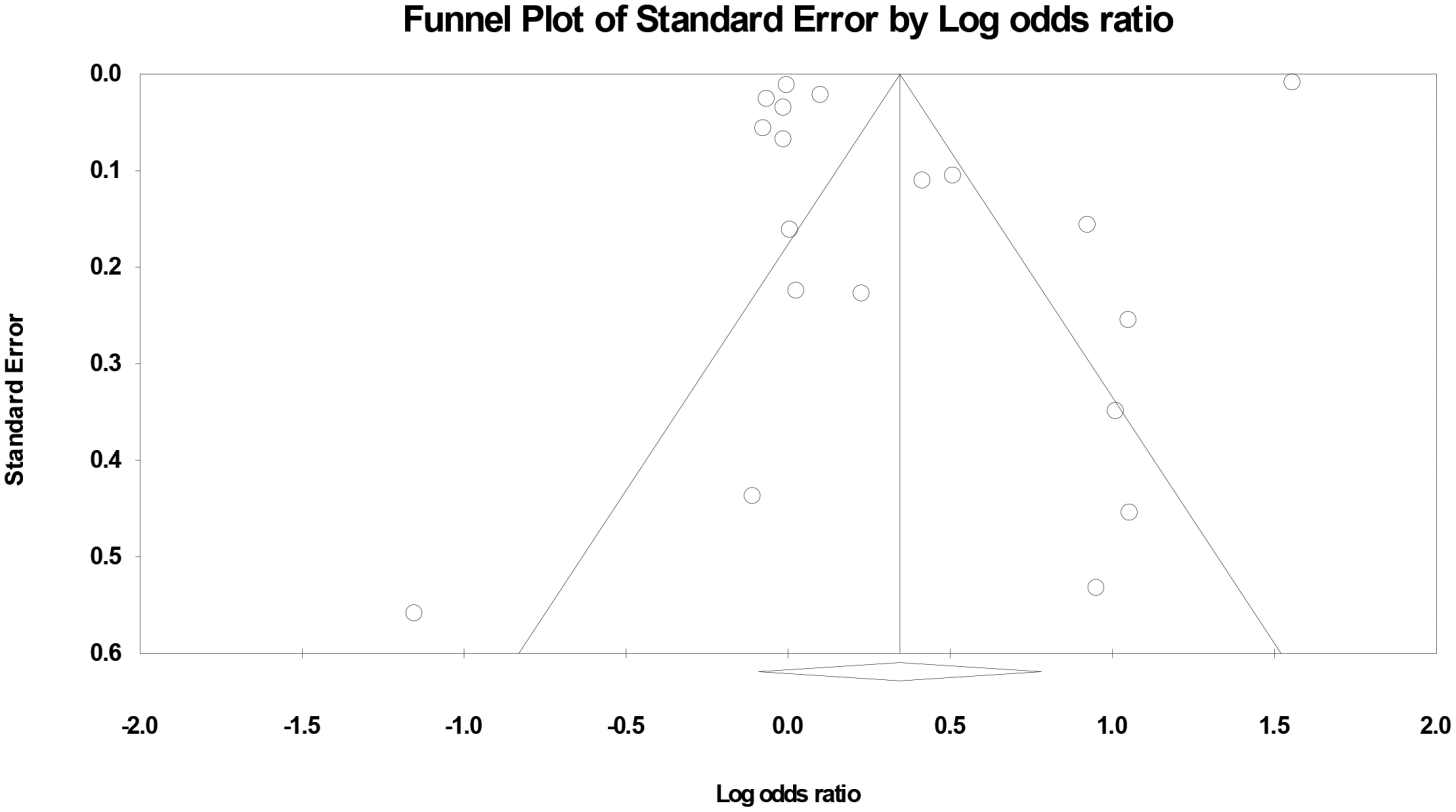
Funnel plot of long working hours and suicidal ideation.

In the association between long working hours and suicidal ideation, the funnel plot is in Figure 21. The Egger test was (*P* = .298), and publication bias was not shown. The trim-and-fill^[55]^ has not been imputed in any study. Heterogeneity was *I^2^* = 99.85%, this is a high- heterogeneity^[123]^ and the heterogeneity of chi-square was 12358.624 (d.f = 19; *P* < .001).

## 4. Discussion

This study employed a systematic review and meta-analysis to investigate occupational factors linked to suicidal ideation, suicide attempts, and suicide mortality. A total of sixty studies met the inclusion criteria, enabling a comprehensive analysis. Sixteen specific occupational risk factors were systematically examined, with their contributions to suicidal ideation, suicide attempts, and suicide mortality thoroughly evaluated.

The findings derived from this research indicate an association between occupational factors and an elevated risk of experiencing suicidal ideations, engaging in suicide attempts, and facing suicide-related mortality. However, it is worth noting that certain relationships observed within the study did not reach a level of statistical significance, suggesting variability in the strength of these associations across different occupational influences. This finding is in line with previous review and meta-analysis studies that investigated some occupational factors, including working conditions ^[41]^ job strain ^[42]^ and workplace bullying.^[39,43]^ With the difference that in past research, they focused on a few occupational risk factors, but in this meta-analysis, a wide set of occupational risk factors related to suicide dimensions were studied. The effect of work and work environment on health is a matter that has been well shown in your previous study.^[27,124]^ Therefore, having a job and doing this type of activity can increase the feeling of mental health^[125]^; also, not having a job and unemployment are related to increasing mental health problems such as depression and suicide.^[22,126]^ When job conditions become unfavorable, it leads a person to be prone to mental and physical health problems.^[127,128]^ On the other hand, improvement in working conditions is associated with improvement in mental health.^[129]^

In general, people of working age spend an important part of their time in the work environment; whether this work environment is a risk factor or a factor that improves mental health depends on the conditions in it. Some mechanisms can be considered through which occupational risk factors affect suicide dimensions. Depression is one of the most important factors affecting the risk of suicide, and therefore, when job conditions are unfavorable, it leads a person to helplessness, fatigue, and depression.^[130–132]^ It can be said that the effect of occupational risk factors on suicide dimensions is mediated through depression. That is, when a person is in an unfavorable job situation, the person becomes depressed, and this depression leads the person to be prone to suicide behaviors. Also, some occupational risk factors, such as being a victim of bullying at work, can lead to psychological trauma for a person, and trauma is a risk factor for suicidal behaviors.^[133]^ Regarding occupational stressors, a previous study has pointed out the role of mental health problems as an important factor in suicide.^[40]^

According to the report published by the WHO, 12 billion working days are lost annually as a result of depression and anxiety, and the resulting economic burden is estimated at 1 trillion.^[134]^ According to that report, almost 60% of the working population is working; therefore, it is necessary to pay attention to effective strategies in reducing the risk of mental health facts at work and also try to follow up on mental problems and their recovery.^[134]^

## 5. Conclusion

This study highlights the significant influence of the work environment and its associated risk factors on various dimensions of suicide. Consequently, workplace health policies must emphasize the importance of monitoring and screening mental health within professional settings. This approach can help identify potential causes contributing to mental health challenges while keeping track of the current situation. Simultaneously, efforts should be directed toward mitigating and reducing these effects. It is important to recognize that suicide-related outcomes are complex and multidimensional, requiring a comprehensive approach. Alongside general monitoring, workplace organizations should implement tailored programs for individuals to actively foster and enhance mental health. To summarize, the findings of the current study highlight the significant impact that occupational factors can have on diminishing mental health. Specifically, this research focuses on the mental health issue of suicide, including its various forms. By gaining a deeper understanding of these workplace-related factors, it becomes possible to play a pivotal role in alleviating the burden of mental health challenges within professional environments.

### 5.1. Limitations

This research provided a perspective on a wide range of occupational risk factors related to suicide dimensions, which can be used in other studies in the field of mental health. However, the current research has limitations. Firstly, it was not possible to investigate gender differences, and considering that suicide has a different prevalence in both genders, this issue needs to be taken into account when generalizing the results. This research analyzed a study that demonstrated considerable heterogeneity. Such pronounced variability diminishes the accuracy of pooled estimates and highlights the inherent challenges in the process. The authors openly recognize this limitation and offer a thoughtful discussion on potential factors contributing to heterogeneity, such as variations in populations, outcome measures, participant characteristics, and study designs. Considering the diverse range of studies included, a certain level of heterogeneity is to be anticipated within this body of literature. Furthermore, the subgroup analyses seem to mitigate concerns related to interoperability to some extent by categorizing outcomes and exposures separately. Most of the studies included in this study focused on suicidal ideation, and suicide attempts and suicide death were less studied. The studies obtained in this research were mostly cross-sectional, so caution should be taken in establishing causal relationships. The plan of suicide and self-harm concerning occupational risk factors had almost no research background; this issue can be investigated in the future. The identified limitations also encompassed the absence of subgroup or meta-regression analyses, the omission of studies employing hazard ratio data, the possible impact of publication bias on the results, and the language-based restrictions applied to the selection of included studies.

## Author contributions

**Conceptualization:** Sohrab Amiri, Moien A.B. Khan.

**Data curation:** Sohrab Amiri.

**Formal analysis:** Sohrab Amiri.

**Writing – original draft:** Sohrab Amiri, Moien A.B. Khan.

**Writing – review & editing:** Sohrab Amiri, Moien A.B. Khan.





## References

[1] Naghavi M; Global Burden of Disease Self-Harm Collaborators. Global, regional, and national burden of suicide mortality 1990 to 2016: systematic analysis for the Global Burden of Disease Study 2016. BMJ. 2019;364:l94. 31339847 10.1136/bmj.l94 PMC6598639

[2] Saxena S Funk MK Chisholm D. Comprehensive mental health action plan 2013–2020. Information for Authors. 1995;1:17. 26442884

[3] Crosby A Ortega L Melanson C. Self-directed violence surveillance; uniform definitions and recommended data elements. 2011. https://www.suicideinfo.ca/resource/self‐directed‐violence‐surveillance‐uniform‐definitions‐and‐recommended‐data‐elements/.

[4] World Health Organization. Preventing Suicide: A Global Imperative. World Health Organization; 2014.

[5] Turecki G Brent DA. Suicide and suicidal behaviour. Lancet. 2016;387:1227–39. 26385066 10.1016/S0140-6736(15)00234-2 PMC5319859

[6] World Health Organization. Suicide. 2023. https://www.who.int/news-room/fact-sheets/detail/suicide. Accessed 2025.

[7] National Institute of Mental Health. Suicide. https://www.nimh.nih.gov/health/statistics/suicide. Accessed 2025.

[8] Cao X-L Zhong B-L Xiang Y-T. Prevalence of suicidal ideation and suicide attempts in the general population of China: a meta-analysis. Int J Psychiatry Med. 2015;49:296–308. 26060259 10.1177/0091217415589306 PMC4536918

[9] WHO. Suicide in the world. 2017. https://www.who.int/news‐room/fact‐sheets/detail/suicide.

[10] Kessler RC Borges G Walters EE. Prevalence of and risk factors for lifetime suicide attempts in the National Comorbidity Survey. Arch Gen Psychiatry. 1999;56:617–26. 10401507 10.1001/archpsyc.56.7.617

[11] Favril L Yu R Uyar A Sharpe M Fazel S. Risk factors for suicide in adults: systematic review and meta-analysis of psychological autopsy studies. Evid Based Ment Health. 2022;25:148–55. 36162975 10.1136/ebmental-2022-300549 PMC9685708

[12] Chesney E Goodwin GM Fazel S. Risks of all-cause and suicide mortality in mental disorders: a meta-review. World Psychiatry. 2014;13:153–60. 24890068 10.1002/wps.20128 PMC4102288

[13] Hoertel N Franco S Wall MM. Mental disorders and risk of suicide attempt: a national prospective study. Mol Psychiatry. 2015;20:718–26. 25980346 10.1038/mp.2015.19

[14] Harris EC Barraclough B. Suicide as an outcome for mental disorders. A meta-analysis. Br J Psychiatry. 1997;170:205–28. 9229027 10.1192/bjp.170.3.205

[15] Bolton JM Walld R Chateau D Finlayson G Sareen J. Risk of suicide and suicide attempts associated with physical disorders: a population-based, balancing score-matched analysis. Psychol Med. 2015;45:495–504. 25032807 10.1017/S0033291714001639

[16] Erlangsen A Stenager E Conwell Y. Physical diseases as predictors of suicide in older adults: a nationwide, register-based cohort study. Soc Psychiatry Psychiatr Epidemiol. 2015;50:1427–39. 25835959 10.1007/s00127-015-1051-0

[17] Qin P Webb R Kapur N Sørensen HT. Hospitalization for physical illness and risk of subsequent suicide: a population study. J Intern Med. 2013;273:48–58. 22775487 10.1111/j.1365-2796.2012.02572.x

[18] Amiri S Behnezhad S. Cancer diagnosis and suicide mortality: a systematic review and meta-analysis. Arch Suicide Res. 2020;24:S94–S112. 30955459 10.1080/13811118.2019.1596182

[19] Amiri S Behnezhad S. Body mass index and risk of suicide: a systematic review and meta-analysis. J Affect Disord. 2018;238:615–25. 29957479 10.1016/j.jad.2018.05.028

[20] Uddin R Burton NW Maple M Khan SR Khan A. Suicidal ideation, suicide planning, and suicide attempts among adolescents in 59 low-income and middle-income countries: a population-based study. Lancet Child Adolesc Health. 2019;3:223–33. 30878117 10.1016/S2352-4642(18)30403-6

[21] Iemmi V Bantjes J Coast E. Suicide and poverty in low-income and middle-income countries: a systematic review. Lancet Psychiatry. 2016;3:774–83. 27475770 10.1016/S2215-0366(16)30066-9

[22] Amiri S. Unemployment and suicide mortality, suicide attempts, and suicide ideation: a meta-analysis. Int J Ment Health. 2022;51:294–318.

[23] Amiri S. Prevalence of suicide in immigrants/refugees: a systematic review and meta-analysis. Arch Suicide Res. 2022;26:370–405. 32780682 10.1080/13811118.2020.1802379

[24] Amiri S Behnezhad S. Alcohol use and risk of suicide: a systematic review and Meta-analysis. J Addict Dis. 2020;38:200–13. 32469287 10.1080/10550887.2020.1736757

[25] Poorolajal J Haghtalab T Farhadi M Darvishi N. Substance use disorder and risk of suicidal ideation, suicide attempt and suicide death: a meta-analysis. J Public Health. 2016;38:e282–91. 10.1093/pubmed/fdv148 26503486

[26] Amiri S Khan MA. Prevalence of non-suicidal self-injury, suicidal ideation, suicide attempts, suicide mortality in eating disorders: a systematic review and meta-analysis. Eat Disord. 2023;31:487–525. 37021980 10.1080/10640266.2023.2196492

[27] Stansfeld S Candy B. Psychosocial work environment and mental health--a meta-analytic review. Scand J Work Environ Health. 2006;32:443–62. 17173201 10.5271/sjweh.1050

[28] WHO. What are social determinants of health? 2012. 2012. https://www.paho.org/en/topics/social-determinants-health. Accessed 2025.

[29] Bonde JP. Psychosocial factors at work and risk of depression: a systematic review of the epidemiological evidence. Occup Environ Med. 2008;65:438–45. 18417557 10.1136/oem.2007.038430

[30] Madsen IEH Nyberg ST Magnusson Hanson LL.; IPD-Work Consortium. Job strain as a risk factor for clinical depression: systematic review and meta-analysis with additional individual participant data. Psychol Med. 2017;47:1342–56. 28122650 10.1017/S003329171600355X PMC5471831

[31] Ahola K Hakanen J. Job strain, burnout, and depressive symptoms: a prospective study among dentists. J Affect Disord. 2007;104:103–10. 17448543 10.1016/j.jad.2007.03.004

[32] Amiri S Behnezhad S. Is job strain a risk factor for musculoskeletal pain? A systematic review and meta-analysis of 21 longitudinal studies. Public Health. 2020;181:158–67. 32059156 10.1016/j.puhe.2019.11.023

[33] Amiri S Behnezhad S. Job strain and mortality ratio: a systematic review and meta-analysis of cohort studies. Public Health. 2020;181:24–33. 31927337 10.1016/j.puhe.2019.10.030

[34] Nielsen MB Harris A Pallesen S Einarsen SV. Workplace bullying and sleep: a systematic review and meta-analysis of the research literature. Sleep Med Rev. 2020;51:101289. 32179375 10.1016/j.smrv.2020.101289

[35] Nielsen MB Indregard AMR Øverland S. Workplace bullying and sickness absence: a systematic review and meta-analysis of the research literature. Scand J Work Environ Health. 2016;42:359–70. 27310716 10.5271/sjweh.3579

[36] Niedhammer I David S Degioanni S. Association between workplace bullying and depressive symptoms in the French working population. J Psychosom Res. 2006;61:251–9. 16880029 10.1016/j.jpsychores.2006.03.051

[37] Gullander M Hogh A Hansen AM. Exposure to workplace bullying and risk of depression. J Occup Environ Med. 2014;56:1258–65. 25479295 10.1097/JOM.0000000000000339

[38] Rodríguez-Muñoz A Moreno-Jiménez B Sanz-Vergel AI. Reciprocal relations between workplace bullying, anxiety, and vigor: a two-wave longitudinal study. Anxiety Stress Coping. 2015;28:514–30. 25665500 10.1080/10615806.2015.1016003

[39] Luo Z Wang J Zhou Y Mao Q Lang B Xu S. Workplace bullying and suicidal ideation and behaviour: a systematic review and meta-analysis. Public Health. 2023;222:166–74. 37544128 10.1016/j.puhe.2023.07.007

[40] Milner A Witt K LaMontagne AD Niedhammer I. Psychosocial job stressors and suicidality: a meta-analysis and systematic review. Occup Environ Med. 2018;75:245–53. 28851757 10.1136/oemed-2017-104531

[41] LaMontagne AD Milner A. Working conditions as modifiable risk factors for suicidal thoughts and behaviours. Occup Environ Med. 2017;74:4–5. 27919064 10.1136/oemed-2016-104036

[42] Niedhammer I Bertrais S Witt K. Psychosocial work exposures and health outcomes: a meta-review of 72 literature reviews with meta-analysis. Scand J Work Environ Health. 2021;47:489–508. 34042163 10.5271/sjweh.3968 PMC8504166

[43] Leach LS Poyser C Butterworth P. Workplace bullying and the association with suicidal ideation/thoughts and behaviour: a systematic review. Occup Environ Med. 2017;74:72–9. 27663985 10.1136/oemed-2016-103726

[44] Çelik S. Cognitive consequences of occupational stress in underground mine workers: neuropsychological observational study. Medicine (Baltimore). 2025;104:e42203. 40258755 10.1097/MD.0000000000042203 PMC12014073

[45] Hughes CL Liu B Qin P. Socioeconomic, demographic, and occupational risk factors for suicide amongst males: a systematic review and meta-analysis. J Affect Disord. 2025;391:119986. 40721142 10.1016/j.jad.2025.119986

[46] Magnusson Hanson LL Madsen IEH Blomqvist S Holmgren R Sørensen K Rugulies R. Employment and working conditions and risk of suicidal behaviors: a systematic review and meta-analysis of longitudinal studies [published online ahead of print June 11, 2026]. Scand J Work Environ Health. doi: 10.5271/sjweh.4308. 10.5271/sjweh.4308 PMC13541624 42274385

[47] Page MJ McKenzie JE Bossuyt PM. The PRISMA 2020 statement: an updated guideline for reporting systematic reviews. BMJ. 2021;372:n71. 33782057 10.1136/bmj.n71 PMC8005924

[48] Armijo-Olivo S Stiles CR Hagen NA Biondo PD Cummings GG. Assessment of study quality for systematic reviews: a comparison of the Cochrane Collaboration Risk of Bias Tool and the Effective Public Health Practice Project Quality Assessment Tool: methodological research. J Eval Clin Pract. 2012;18:12–8. 20698919 10.1111/j.1365-2753.2010.01516.x

[49] Thomas H. Quality assessment tool for quantitative studies. In: Effective Public Health Practice Project. 2003. https://www.ephpp.ca/quality‐assessment‐tool‐for‐quantitative‐studies/

[50] Borenstein M Hedges LV Higgins JPT Rothstein HR. Introduction to Meta-Analysis. John Wiley & Sons; 2021.

[51] Higgins JP Thompson SG. Quantifying heterogeneity in a meta-analysis. Stat Med. 2002;21:1539–58. 12111919 10.1002/sim.1186

[52] Ioannidis JP Patsopoulos NA Evangelou E. Uncertainty in heterogeneity estimates in meta-analyses. BMJ. 2007;335:914–6. 17974687 10.1136/bmj.39343.408449.80 PMC2048840

[53] Begg CB Mazumdar M. Operating characteristics of a rank correlation test for publication bias. Biometrics. 1994;50:1088–101. 7786990

[54] Egger M Davey Smith G Schneider M Minder C. Bias in meta-analysis detected by a simple, graphical test. BMJ. 1997;315:629–34. 9310563 10.1136/bmj.315.7109.629 PMC2127453

[55] Duval S Tweedie R. Trim and fill: a simple funnel-plot-based method of testing and adjusting for publication bias in meta-analysis. Biometrics. 2000;56:455–63. 10877304 10.1111/j.0006-341x.2000.00455.x

[56] Sterne JAC Sutton AJ Ioannidis JPA. Recommendations for examining and interpreting funnel plot asymmetry in meta-analyses of randomised controlled trials. BMJ. 2011;343:d4002. 21784880 10.1136/bmj.d4002

[57] Borenstein M Hedges L Higgins J Rothstein H. Comprehensive Meta-Analysis Version 3.3. 070. Biostat; 2014:104.

[58] Almroth M Hemmingsson T Kjellberg K. Job control, job demands and job strain and suicidal behaviour among three million workers in Sweden. Occup Environ Med. 2022;79:681–9. 35803712 10.1136/oemed-2022-108268 PMC9484393

[59] Baumert J Schneider B Lukaschek K. Adverse conditions at the workplace are associated with increased suicide risk. J Psychiatr Res. 2014;57:90–5. 25012186 10.1016/j.jpsychires.2014.06.007

[60] Blomqvist S Virtanen M LaMontagne AD Magnusson Hanson LL. Perceived job insecurity and risk of suicide and suicide attempts: a study of men and women in the Swedish working population. Scand J Work Environ Health. 2022;48:293–301. 35184203 10.5271/sjweh.4015 PMC9524162

[61] Kim M-S Hong Y-C Yook J-H Kang M-Y. Effects of perceived job insecurity on depression, suicide ideation, and decline in self-rated health in Korea: a population-based panel study. Int Arch Occup Environ Health. 2017;90:663–71. 28509941 10.1007/s00420-017-1229-8

[62] Niedhammer I Chastang J-F Coutrot T Geoffroy-Perez B LaMontagne AD Milner A. Psychosocial work exposures of the job strain model and suicide in France: findings from the STRESSJEM prospective study of 1.5 million men and women over 26 years of follow-up. Psychother Psychosom. 2020;89:398–401. 32203967 10.1159/000506713

[63] Ahn SH Lee YJ Jang EC Kwon SC Min YS Ryu SH. A study of job stress, suicidal ideation and suicide attempts in display manufacturing workers: a cross-sectional study. Ann Occup Environ Med. 2020;32:16. 10.35371/aoem.2020.32.e16 PMC7332350 32676194

[64] Akram B Bibi B Ashfaq Ahmed M Kausar N. Work-family conflict and suicidal ideation among physicians of Pakistan: the moderating role of perceived life satisfaction. Omega (Westport). 2022;85:465–82. 32762292 10.1177/0030222820947246

[65] Andela M. Burnout, somatic complaints, and suicidal ideations among veterinarians: development and validation of the veterinarians stressors inventory. J Vet Behav. 2020;37:48–55.

[66] Atay IM Eren I GündoğAr D. The prevalence of death ideation and attempted suicide and the associated risk factors in Isparta, Turkey. Turk J Psychiatry. 2012;23:89–98. 22648871

[67] Begashaw T Kassie G. Prevalence and associated factors of suicidal ideation among Almeda Textile Factory workers in Adwa, Tigray, Ethiopia: a cross-sectional study. Biomed Res Int. 2022;2022:9459186. 36588536 10.1155/2022/9459186 PMC9800075

[68] Berg AM Hem E Lau B Ekeberg O. An exploration of job stress and health in the Norwegian police service: a cross sectional study. J Occup Med Toxicol. 2006;1:26. 17156489 10.1186/1745-6673-1-26 PMC1764750

[69] Byun J Kim H-R Lee H-E Kim S-E Lee J. Factors associated with suicide ideation among subway drivers in Korea. Ann Occup Environ Med. 2016;28:1–6. 27486516 10.1186/s40557-016-0120-5 PMC4969654

[70] Campbell-Sills L Sun X Kessler RC Ursano RJ Jain S Stein MB. Exposure to bullying or hazing during deployment and mental health outcomes among US Army soldiers. JAMA Netw Open. 2023;6:e2252109. 36692883 10.1001/jamanetworkopen.2022.52109 PMC10408263

[71] Chin WS Chen Y-C Ho J-J Cheng N-Y Wu H-C Shiao JSC. Psychological work environment and suicidal ideation among nurses in Taiwan. J Nurs Scholarsh. 2019;51:106–13. 30466180 10.1111/jnu.12441

[72] Chireh B Essien SK Novik N Ankrah M. Long working hours, perceived work stress, and common mental health conditions among the full-time Canadian working population: a national comparative study. J Affect Disord Rep. 2023;12:100508.

[73] Choi B. Job strain, long work hours, and suicidal ideation in US workers: a longitudinal study. Int Arch Occup Environ Health. 2018;91:865–75. 29959524 10.1007/s00420-018-1330-7

[74] Dalglish SL Melchior M Younes N Surkan PJ. Work characteristics and suicidal ideation in young adults in France. Soc Psychiatry Psychiatr Epidemiol. 2015;50:613–20. 25308058 10.1007/s00127-014-0969-y

[75] Dalum HS Tyssen R Hem E. Prevalence and individual and work-related factors associated with suicidal thoughts and behaviours among veterinarians in Norway: a cross-sectional, nationwide survey-based study (the NORVET study). BMJ Open. 2022;12:e055827. 10.1136/bmjopen-2021-055827 PMC8724721 34980627

[76] Dias AR Fernandes SM Fialho-Silva I Cerqueira-Silva T Miranda-Scippa A Almeida AG. Burnout syndrome and resilience in medical students from a Brazilian public college in Salvador, Brazil. Trends Psychiatry Psychother. 2022;44:e20200187. 34139116 10.47626/2237-6089-2020-0187 PMC9907392

[77] Dyrbye LN Thomas MR Massie FS. Burnout and suicidal ideation among US medical students. Ann Intern Med. 2008;149:334–41. 18765703 10.7326/0003-4819-149-5-200809020-00008

[78] Ejdesgaard BA Zøllner L Jensen BF Jørgensen H-O Kähler H. Risk and protective factors for suicidal ideation and suicide attempts among deployed Danish soldiers from 1990 to 2009. Mil Med. 2015;180:61–7. 10.7205/MILMED-D-13-00353 25562859

[79] Feskanich D Hastrup JL Marshall JR. Stress and suicide in the Nurses’ Health Study. J Epidemiol Community Health. 2002;56:95–8. 11812806 10.1136/jech.56.2.95 PMC1732078

[80] Fresán A Yoldi-Negrete M Robles-García R Tovilla-Zárate C-A Suárez-Mendoza A. Professional adversities and protective factors associated with suicidal ideation in Mexican psychiatrists. Arch Med Res. 2019;50:484–9. 32018070 10.1016/j.arcmed.2019.11.010

[81] Han S Ko Y Moon JE Cho YS. Working hours are closely associated with depressive mood and suicidal ideation in Korean adults: a nationwide cross-sectional study. Sci Rep. 2021;11:23102. 34845294 10.1038/s41598-021-02574-8 PMC8630033

[82] Ishikawa M. Relationships between overwork, burnout and suicidal ideation among resident physicians in hospitals in Japan with medical residency programmes: a nationwide questionnaire-based survey. BMJ Open. 2022;12:e056283. 10.1136/bmjopen-2021-056283 PMC8915267 35273058

[83] Ishikawa M. Long working hours, depression and suicidality among OB/GYNs in Japan. Occup Med (Oxf). 2022;72:200–6. 35064662 10.1093/occmed/kqab191

[84] Jesus A Pitacho L Moreira A. Burnout and suicidal behaviours in health professionals in Portugal: the moderating effect of self-esteem. Int J Environ Res Public Health. 2023;20:4325. 36901334 10.3390/ijerph20054325 PMC10002387

[85] Kang M-Y Kwon H-J Choi K-H Kang C-W Kim H. The relationship between shift work and mental health among electronics workers in South Korea: a cross-sectional study. PLoS One. 2017;12:e0188019. 29145440 10.1371/journal.pone.0188019 PMC5690616

[86] Kim S-Y Shin D-W Oh K-S. Gender differences of occupational stress associated with suicidal ideation among South Korean employees: the Kangbuk Samsung health study. Psychiatry Investig. 2018;15:156–63. 10.30773/pi.2017.05.31.1 PMC5900399 29475218

[87] Kyron MJ Rikkers W Page AC. Prevalence and predictors of suicidal thoughts and behaviours among Australian police and emergency services employees. Aust N Z J Psychiatry. 2021;55:180–95. 32615800 10.1177/0004867420937774

[88] Langhinrichsen-Rohling J Snarr JD Slep AMS Heyman RE Foran HM; United States Air Force Family Advocacy Program. Risk for suicidal ideation in the US Air Force: an ecological perspective. J Consult Clin Psychol. 2011;79:600–12. 21787046 10.1037/a0024631

[89] Leach LS Too LS Batterham PJ Kiely KM Christensen H Butterworth P. Workplace bullying and suicidal ideation: findings from an Australian longitudinal cohort study of mid-aged workers. Int J Environ Res Public Health. 2020;17:1448. 32102336 10.3390/ijerph17041448 PMC7068571

[90] Li Z Liu D Liu X Su H Bai S. The association of experienced long working hours and depression, anxiety, and suicidal ideation among Chinese medical residents during the COVID-19 pandemic: a multi-center cross-sectional study. Psychol Res Behav Manag. 2023;16:1459–70. 37131958 10.2147/PRBM.S408792 PMC10149078

[91] Lin W Wang H Gong L. Work stress, family stress, and suicide ideation: a cross-sectional survey among working women in Shenzhen, China. J Affect Disord. 2020;277:747–54. 32919296 10.1016/j.jad.2020.08.081

[92] Loerbroks A Cho S-I Dollard MF. Associations between work stress and suicidal ideation: individual-participant data from six cross-sectional studies. J Psychosom Res. 2016;90:62–9. 27772561 10.1016/j.jpsychores.2016.09.008

[93] Lu YE Sun M Li Y. Association of workplace bullying with suicide ideation and attempt among Chinese nurses during the COVID-19 pandemic. J Clin Psychol Med Settings. 2022;30:687–96. 36272037 10.1007/s10880-022-09915-3 PMC9589744

[94] Menon NK Shanafelt TD Sinsky CA. Association of physician burnout with suicidal ideation and medical errors. JAMA Netw Open. 2020;3:e2028780. 33295977 10.1001/jamanetworkopen.2020.28780 PMC7726631

[95] Milner A Currier D LaMontagne AD Spittal MJ Pirkis J. Psychosocial job stressors and thoughts about suicide among males: a cross-sectional study from the first wave of the Ten to Men cohort. Public Health. 2017;147:72–6. 28404500 10.1016/j.puhe.2017.02.003

[96] Milner A Page K Witt K LaMontagne A. Psychosocial working conditions and suicide ideation. J Occup Environ Med. 2016;58:584–7. 27206117 10.1097/JOM.0000000000000700

[97] Milner A Spittal MJ Pirkis J Chastang J-F Niedhammer I LaMontagne AD. Low control and high demands at work as risk factors for suicide: an Australian national population-level case-control study. Psychosom Med. 2017;79:358–64. 27580270 10.1097/PSY.0000000000000389

[98] Mo PKH Cheng Y Lau JTF. Work‐related factors on mental health among migrant factory workers in China: application of the demand‐control and effort‐reward imbalance model. Health Soc Care Community. 2022;30:656–67. 32989898 10.1111/hsc.13176

[99] Nie G Du J Liu J Yuan L Ma Z. Job stress and suicidal ideation among Chinese clinicians: the moderating role of social support. J Gen Psychol. 2020;147:109–22. 31318662 10.1080/00221309.2019.1640657

[100] Niedhammer I Bèque M Chastang J-F Bertrais S. Psychosocial work exposures and suicide ideation: a study of multiple exposures using the French national working conditions survey. BMC Public Health. 2020;20:1–10. 32517747 10.1186/s12889-020-09019-3 PMC7285589

[101] Nielsen MB Einarsen S Notelaers G Nielsen GH. Does exposure to bullying behaviors at the workplace contribute to later suicidal ideation? A three-wave longitudinal study. Scand J Work Environ Health. 2016;42:246–50. 27135593 10.5271/sjweh.3554

[102] Ostry A Maggi S Tansey J. The impact of psychosocial work conditions on attempted and completed suicide among Western Canadian sawmill workers. Scand J Public Health. 2007;35:265–71. 17530548 10.1080/14034940601048091

[103] Park H Kim JI Min B Oh S Kim J-H. Prevalence and correlates of suicidal ideation in Korean firefighters: a nationwide study. BMC Psychiatry. 2019;19:1–9. 31888659 10.1186/s12888-019-2388-9 PMC6937629

[104] Park H Suh BS Lee K. The difference of suicidal ideation between shift workers and day workers by gender. Arch Suicide Res. 2022;26:928–36. 33211634 10.1080/13811118.2020.1847708

[105] Park S Kook H Seok H. The negative impact of long working hours on mental health in young Korean workers. PLoS One. 2020;15:e0236931. 32750080 10.1371/journal.pone.0236931 PMC7402483

[106] Petrie K Crawford J LaMontagne AD. Working hours, common mental disorder and suicidal ideation among junior doctors in Australia: a cross-sectional survey. BMJ Open. 2020;10:e033525. 10.1136/bmjopen-2019-033525 PMC7045753 31964674

[107] Petrie K Crawford J Shand F Harvey SB. Workplace stress, common mental disorder and suicidal ideation in junior doctors. Intern Med J. 2021;51:1074–80. 33135841 10.1111/imj.15124 PMC8362052

[108] Ross DV Mathieu DS Wardhani MR Gullestrup MJ Kõlves DK. Suicidal ideation and related factors in construction industry apprentices. J Affect Disord. 2022;297:294–300. 34710501 10.1016/j.jad.2021.10.073

[109] Son J Lee S. Effects of work stress, sleep, and shift work on suicidal ideation among female workers in an electronics company. Am J Ind Med. 2021;64:519–27. 33749856 10.1002/ajim.23243

[110] Stanley IH Boffa JW Smith LJ. Occupational stress and suicidality among firefighters: examining the buffering role of distress tolerance. Psychiatry Res. 2018;266:90–6. 29857292 10.1016/j.psychres.2018.05.058 PMC6397653

[111] Sterud T Hem E Lau B Ekeberg O. Suicidal ideation and suicide attempts in a nationwide sample of operational Norwegian ambulance personnel. J Occup Health. 2008;50:406–14. 18654042 10.1539/joh.l8025

[112] Sugawara N Yasui-Furukori N Sasaki G. Gender differences in factors associated with suicidal ideation and depressive symptoms among middle-aged workers in Japan. Ind Health. 2013;51:202–13. 23268835 10.2486/indhealth.ms1354

[113] Takada M Suzuki A Shima S Inoue K Kazukawa S Hojoh M. Associations between lifestyle factors, working environment, depressive symptoms and suicidal ideation: a large-scale study in Japan. Ind Health. 2009;47:649–55. 19996541 10.2486/indhealth.47.649

[114] Takeuchi A Sakano N Miyatake N. Combined effects of working hours, income, and leisure time on suicide in all 47 prefectures of Japan. Ind Health. 2014;52:137–40. 24464025 10.2486/indhealth.2013-0182 PMC4202760

[115] Takusari E Suzuki M Nakamura H Otsuka K. Mental health, suicidal ideation, and related factors among workers from medium-sized business establishments in northern Japan: comparative study of sex differences. Ind Health. 2011;49:452–63. 21697623 10.2486/indhealth.ms1251

[116] Tsuno K Tabuchi T. Risk factors for workplace bullying, severe psychological distress and suicidal ideation during the COVID-19 pandemic among the general working population in Japan: a large-scale cross-sectional study. BMJ Open. 2022;12:e059860. 10.1136/bmjopen-2021-059860 PMC9638740 36323475

[117] Tsutsumi A Kayaba K Ojima T Ishikawa S Kawakami N; Jichi Medical School Cohort Study Group. Low control at work and the risk of suicide in Japanese men: a prospective cohort study. Psychother Psychosom. 2007;76:177–85. 17426417 10.1159/000099845

[118] Tyssen R Vaglum P Grønvold NT Ekeberg O. Suicidal ideation among medical students and young physicians: a nationwide and prospective study of prevalence and predictors. J Affect Disord. 2001;64:69–79. 11292521 10.1016/s0165-0327(00)00205-6

[119] Wall M Schenck-Gustafsson K Minucci D Sendén MG Løvseth LT Fridner A. Suicidal ideation among surgeons in Italy and Sweden–a cross-sectional study. BMC Psychol. 2014;2:1–8. 10.1186/s40359-014-0053-0 PMC4266411 25520811

[120] Wang XL Yip PSF Chan CLW. Suicide prevention for local public and volunteer relief workers in disaster-affected areas. J Public Health Manag Pract. 2016;22:E39–46. 23760310 10.1097/PHH.0b013e31829a303c

[121] Yoon J-H Jeung D Chang S-J. Does high emotional demand with low job control relate to suicidal ideation among service and sales workers in Korea? J Korean Med Sci. 2016;31:1042–8. 27366000 10.3346/jkms.2016.31.7.1042 PMC4900994

[122] Younes N Rivière M Plancke L. Work intensity in men and work-related emotional demands in women are associated with increased suicidality among persons attending primary care. J Affect Disord. 2018;235:565–73. 29698918 10.1016/j.jad.2018.04.075

[123] Higgins JP Thompson SG Deeks JJ Altman DG. Measuring inconsistency in meta-analyses. BMJ. 2003;327:557–60. 12958120 10.1136/bmj.327.7414.557 PMC192859

[124] Belloni M Carrino L Meschi E. The impact of working conditions on mental health: novel evidence from the UK. Lab Econ. 2022;76:102176.

[125] Marmot M Friel S Bell R Houweling TAJ Taylor S; Commission on Social Determinants of Health. Closing the gap in a generation: health equity through action on the social determinants of health. Lancet. 2008;372:1661–9. 18994664 10.1016/S0140-6736(08)61690-6

[126] Amiri S. Unemployment associated with major depression disorder and depressive symptoms: a systematic review and meta-analysis. Int J Occup Saf Ergon. 2022;28:2080–92. 34259616 10.1080/10803548.2021.1954793

[127] Barnay T. Health, work and working conditions: a review of the European economic literature. Eur J Health Econ. 2016;17:693–709. 26280132 10.1007/s10198-015-0715-8

[128] Harvey SB Modini M Joyce S. Can work make you mentally ill? A systematic meta-review of work-related risk factors for common mental health problems. Occup Environ Med. 2017;74:301–10. 28108676 10.1136/oemed-2016-104015

[129] Egan M Bambra C Thomas S Petticrew M Whitehead M Thomson H. The psychosocial and health effects of workplace reorganisation. 1. A systematic review of organisational-level interventions that aim to increase employee control. J Epidemiol Community Health. 2007;61:945–54. 17933951 10.1136/jech.2006.054965 PMC2465601

[130] Czuba KJ Kayes NM McPherson KM. Support workers’ experiences of work stress in long-term care settings: a qualitative study. Int J Qual Stud Health Well-Being. 2019;14:1622356. 31156047 10.1080/17482631.2019.1622356 PMC6566720

[131] Norrman Harling M Högman E Schad E. Breaking the taboo: eight Swedish clinical psychologists’ experiences of compassion fatigue. Int J Qual Stud Health Well-Being. 2020;15:1785610. 32631158 10.1080/17482631.2020.1785610 PMC7482789

[132] Burgard SA Elliott MR Zivin K House JS. Working conditions and depressive symptoms: a prospective study of US adults. J Occup Environ Med. 2013;55:1007–14. 24013657 10.1097/JOM.0b013e3182a299af PMC3951142

[133] Sabawoon A Keyes KM Karam E Kovess-Masfety V. Associations between traumatic event experiences, psychiatric disorders, and suicidal behavior in the general population of Afghanistan: findings from Afghan National Mental Health Survey. INJ Epidemiol. 2022;9:1–17. 36203184 10.1186/s40621-022-00403-8 PMC9535941

[134] WHO. Mental health at work. 2022. https://www.who.int/news-room/fact-sheets/detail/mental-health-at-work. Accessed 2025.

