# Supplementary material for: Occupational risk factors for suicide: A systematic review and meta-analysis
Source: Medicine (Baltimore). 2026 Aug 7;105(32):e49903. doi: 10.1097/MD.0000000000049903 (PMC13456902; doi:10.1097/MD.0000000000049903)
Supplement: Supplementary file 1 [file medi-105-e49903-s001.docx]

**Supplemental Digital Content:** Keywords used for PubMed, Scopus, and Web of Science search until August, 2023.

| Search | Query |
| --- | --- |
| PubMed | 20,737 |
| #1 | Suicide [Mesh] OR Suicide [Text Word] OR Suicidal Ideation [Mesh] OR Suicidal Ideation [Text Word] OR Suicide Attempted [Mesh] OR Suicide Attempted [Text Word] OR Suicide Attempts [Text Word] OR Suicide thoughts [Text Word] OR Suicide Plan [Text Word] OR Suicide Behavior [Text Word] OR Self-Injurious Behavior [Mesh] OR Self-Injurious Behavior [Text Word] OR Death wishes [Text Word] OR Self Harm [Text Word] OR Self Injury [Text Word] OR Self-Destructive [Text Word] |
| #2 | Job Strain [Text Word] OR Job demands [Text Word] OR job control [Text Word] OR work strain [Text Word] OR Occupational Stresses [Mesh] OR Occupational Stresses [Text Word] OR Job Stress [Mesh] OR Job Stress [Text Word] OR Professional Stress [Mesh] OR Professional Stress [Text Word] OR Workplace Stress [Mesh] OR Workplace Stress [Text Word] OR Work long hours [Text Word] OR Long working hours [Text Word] OR working hours [Text Word] OR Heavy overtime work [Text Word] OR Overtime work [Text Word] OR Occupational Factors [Text Word] OR Work Conditions [Text Word] OR Risk Factors [Text Word] OR Occupational Stress [Mesh] OR Occupational Stress [Text Word] OR Workplace Bullying [Text Word] OR Job-related [Text Word] OR Occupational environment [Text Word] OR Professional environment [Text Word] OR Workplace Abuses [Text Word] OR Workplace [Text Word] OR effort reward imbalance [Text Word] OR Shift Work Schedule [Mesh] OR Shift Work Schedule [Text Word] OR Shift Work [Text Word] OR Work-Life Balance [Text Word] OR Work Life Conflict [Text Word] OR Work Family Balance [Text Word] OR Workload [Mesh] OR Workload [Text Word] OR Job Satisfaction [Mesh] OR Job Satisfaction [Text Word] OR Working Conditions [Mesh] OR Working Conditions [Text Word] OR Occupational Injuries [Mesh] OR Occupational Injuries [Text Word] OR Occupational Exposure [Mesh] OR Occupational Exposure [Text Word] OR organizational justice[Text Word] OR workplace justice [Text Word] OR decision authority [Text Word] OR job insecurity [Text Word] OR job seniority [Text Word] OR specialization [Text Word] OR work demands [Text Word] OR supervisor support [Text Word] OR Burnout, Psychological [Mesh] OR Burnout, Psychological [Text Word] OR Psychological Burnout [Text Word] OR Burnout Syndrome [Text Word] OR Burnout, Professional [Text Word] OR Burnout, Professional [Text Word] OR Occupational Burnout [Text Word] OR Career Burnout [Text Word] OR emotional exhaustion [Text Word] OR physical exhaustion [Text Word] OR Job burnout [Text Word] OR Depersonalisation [Mesh] OR Depersonalisation [Text Word] |
| Final | #1 AND #2 |
| Scopus | 31,626 |
| #1 | "Suicide" OR "Suicidal Ideation" OR "Suicide Attempted" OR "Suicide Attempts" OR "Suicide thoughts" OR "Suicide Plan" OR "Suicide Behavior" OR "Self-Injurious Behavior" OR "Death wishes" OR "Self Harm" OR "Self Injury" OR "Self-Destructive" |
| #2 | "Job Strain" OR "Job demands" OR "job control" OR "work strain" OR "Occupational Stresses" OR "Occupational Stresses" OR "Job Stress" OR "Professional Stress" OR "Workplace Stress" OR "Work long hours" OR "Long working hours" OR "working hours" OR "Heavy overtime work" OR "Overtime work" OR "Occupational Factors" OR "Work Conditions" OR "Risk Factors" OR "Occupational Stress" OR "Workplace Bullying" OR "Job-related" OR "Occupational environment" OR "Professional environment" OR "Workplace Abuses" OR "Workplace" OR "effort reward imbalance" OR "Shift Work Schedule" OR "Shift Work" OR "Work-Life Balance" OR "Work Life Conflict" OR "Work Family Balance" OR "Workload" OR "Job Satisfaction" OR "Working Conditions" OR "Occupational Injuries" OR "Occupational Exposure" OR "organizational justice" OR "workplace justice" OR "decision authority" OR "job insecurity" OR "specialization" OR "work demands" OR "supervisor support" OR "Burnout, Psychological" OR "Psychological Burnout" OR "Burnout Syndrome" OR "Burnout, Professional" OR "Occupational Burnout" OR "Career Burnout" OR "Emotional exhaustion" OR "physical exhaustion" OR "Job burnout" OR "Depersonalisation" |
| Final | 1 AND #2 |
| Web of Science | 34,234 |
| #1 | TS=(Suicide OR Suicidal Ideation OR Suicide Attempted OR Suicide Attempts OR Suicide thoughts OR Suicide Plan OR Suicide Behavior OR Self-Injurious Behavior OR Death wishes OR Self Harm OR Self Injury OR Self-Destructive) |
| #2 | TS=(Job Strain OR Job demands OR job control OR work strain OR Occupational Stresses OR Occupational Stresses OR Job Stress OR Professional Stress OR Workplace Stress OR Work long hours OR Long working hours OR working hours OR Heavy overtime work OR Overtime work OR Occupational Factors OR Work Conditions OR Risk Factors OR Occupational Stress OR Workplace Bullying OR Job-related OR Occupational environment OR Professional environment OR Workplace Abuses OR Workplace OR effort reward imbalance OR Shift Work Schedule OR Shift Work OR Work-Life Balance OR Work Life Conflict OR Work Family Balance OR Workload OR Job Satisfaction OR Working Conditions OR Occupational Injuries OR Occupational Exposure OR organizational justice OR workplace justice OR decision authority OR job insecurity OR specialization OR work demands OR supervisor support OR Burnout, Psychological OR Psychological Burnout OR Burnout Syndrome OR Burnout, Professional OR Occupational Burnout OR Career Burnout OR Emotional exhaustion OR physical exhaustion OR Job burnout OR Depersonalisation) |
| Final | 1 AND #2 |
