# Supplementary material for: Occupational risk factors for suicide: A systematic review and meta-analysis
Source: Medicine (Baltimore). 2026 Aug 7;105(32):e49903. doi: 10.1097/MD.0000000000049903 (PMC13456902; doi:10.1097/MD.0000000000049903)
Supplement: Supplementary file 2 [file medi-105-e49903-s002.docx]

| First author and year of publication | Country | Population | Study design | Age | Sex  % women | Sample size | Occupational risk factors | Suicide | Outcome Measure | Quality assessment | | | | Results  odd ratio and 95% confidence interval | Adjusted for |
| --- | --- | --- | --- | --- | --- | --- | --- | --- | --- | --- | --- | --- | --- | --- | --- |
|  |  |  |  |  |  |  |  |  |  | Selection | Confounders | Data collection method | Withdrawals and dropouts |  |  |
| Ahn 2020 | Korea | Workers | Cross-sectional | 19-39 | 20.2% women | 836 | 1-Job demand  2-job control  3-Interpersonal conflict  3- Job insecurity  5- Lack of reward  6-Occupational climate | 1-Suicidal ideation  2-Suicide attempts | 1-self-reported questions | Low | Low | Moderate | Low | Odd ratio  Men  Job demand  0.73 (CI 0.17–3.24)  job control  1.14 (CI 0.39–3.32)  Interpersonal conflict  1.30 (CI 0.53–3.20)  Job insecurity  2.35 (CI 0.96–5.74)  Lack of reward  0.68 (CI 0.16–2.92)  Occupational climate  2.00 (CI 0.72–5.51)  Women  Job demand  3.23 (CI 0.67–15.61)  job control  2.45 (CI 0.44–13.81)  Interpersonal conflict  1.83 (CI 0.38–8.85)  Job insecurity  1.74 (CI 0.54–5.58)  Lack of reward  5.31 (CI 1.54–18.34)  Occupational climate  7.36 (CI 2.28–23.72) | age, BMI, smoking, alcohol consumption, regular exercise,  chronic disease, shift work, job tenure, CES-D |
| Akram 2022 | Pakistan | Physicians | Cross-sectional | 32–55 | 46% women | 622 | 1-Work-family conflict | 1-Suicidal ideation | 1-Suicidal Ideation Attribution Scale | Low | High | Low | Low | r  622 (0.41) | Unadjusted |
| Almroth 2022 | Sweden | Workers | Longitudinal | 30–60 at baseline | Both % women not reported | 3million | 1- job control  2- Job demand  3-job strain | 1-suicide attempts  2-suicide mortality | 1-inpatient register  2-cause of death register | Low | Low | Low | Low | Hazard ratio***  Suicide attempts  Men  job control  1.40 (CI 1.27–1.55)  Job demand  0.89 (CI 0.82–0.97)  job strain  1.29 (CI 1.18–1.41)  Suicide mortality  job control  1.36 (CI 1.19–1.55)  Job demand  0.83 (CI 0.74–0.93)  job strain  1.16 (CI 1.03–1.31)  Women  Suicide attempts  job control  1.39 (CI 1.28–1.51)  Job demand  0.87 (CI 0.81–0.95)  job strain  1.06 (CI 1.15–1.30)  Suicide mortality  job control  1.26 (CI 1.04–1.53)  Job demand  1.02 (CI 0.85–1.22)  job strain  1.04 (CI 0.87–1.26) | "age, birthyear, birth country, education, civil status at baseline, children at baseline, previous suicide attempt, previous psychiatric diagnosis, parents’ SEP,  parents’ psychiatric diagnosis, long-term unemployment prior to baseline, long-term sick leave prior to baseline, getting divorced during the follow-up period, having children  during the follow-up period, long term unemployment during the follow-up period and long-term sick leave during the follow-up period" |
| Andela 2020 | France | Veterinarians | Cross-sectional | 18-66 | 72.1% women | 490 | 1- Burnout  2-Work-family conflicts  3-Workload  4-Emotional demands | 1-Suicidal ideation | 1-Garrison scale | Low | High | Low | Low | r  Emotional exhaustion  490 (0.40)  Cynicism  490 (0.38)  Work-family conflicts  490 (0.26)  Workload  490 (0.25)  Emotional demands  490 (0.17) | Unadjusted |
| Atay 2012 | Turkey | General population | Cross-sectional | 18- 65 | 56% women | 600 | 1-Job satisfaction | 1-Suicidal ideation | 1-two item | Low | High | Moderate | Low | Odd ratio**  1.26 (CI 0.78-2.05) | Unadjusted |
| Baumert 2014 | Germany | General population | Longitudinal | 25-74 | 37.4% women | 6,817 | 1-Job strain | 1-Suicide mortality | 1-Death certificates | Moderate | Low | Low | Low | Hazard ratio***  0.82 (CI 0.36-1.84) | age, sex, survey, living alone, low educational level, smoking, high alcohol consumption, obesity, and depressed mood |
| Begashaw 2022 | Ethiopia | Workers | Cross-sectional | ≥18 | 63.6% women | 409 | 1-Long working hours  2-Workload  3-Conflict at work  4-Job dis satisfaction | 1-Suicidal ideation | 1-one item | Low | Moderate | Moderate | Low | Odd ratio  Working hour  2.88 (CI 1.18-7.04)  Workload  1.11 (CI 0.43-2.88)  Conflict at work  3.54 (CI 1.40-8.90)  Job dissatisfaction  0.62 (CI 0.22-1.74) | Adjusted (factors are unknown) |
| Berg 2006 | Norway | Police | Cross-sectional | 20-59 | 15.7% women | 3,272 | 1- Job Stress | 1-Suicidal ideation | 1-Paykel's Suicidal  Feelings | Moderate | Low | Low | Low | Odd ratio  0.8 (CI 0.6-1.19)  1.3 (CI 0.9-1.7) | age, gender, personality, and the other health variables |
| Blomqvist 2022 | Sweden | Employees | Longitudinal | 16-64 | 53% women | 65,571 | 1-job insecurity | 1-suicide mortality  2-suicide attempts | 1-Swedish Patient Register | Low | Moderate | Low | Low | Hazard ratio***  suicide mortality  1.49 (CI 1.01–2.20)  suicide attempts  1.03 (CI 0.86–1.24)  Men  suicide mortality  1.29 (CI 0.77–2.17)  suicide attempts  1.90 (CI 0.97–3.73)  Women  suicide mortality  1.20 (CI 0.93–1.54)  suicide attempts  0.88 (CI 0.68–1.14) | sex, birth country, family situation, educational level |
| Byun 2016 | Korea | Drivers | Cross-sectional | Unknown | Men | 980 | 1-Job demand  2- job control  3-interpersonal conflict  4-Job insecurity  5- Lack of reward  6-Occupational climate | 1-Suicidal ideation | 1-Composite International Diagnostic Interview | Low | Low | Low | Low | Odd ratio  Job demand  1.97 (CI 0.88–4.57)  job control  2.21 (CI 1.01–4.73)  Interpersonal conflict  1.71 (CI 0.70-4.78)  Job insecurity  1.00 (CI 0.25–7.13)  Lack of reward  2.23 (CI 1.02–5.15)  Occupational climate  1.32 (0.59–3.09) | age, marital status, MDD, PTSD, GAD, panic  disorder |
| Campbell sills 2023 | USA | Army Soldiers | Longitudinal | 21.1±0.1 | 9.6% women | 1,463 | 1-Workplace bullying | 1-Suicidal ideation | 1-Composite International Diagnostic Interview  Screening Scales | High | Low | Low | Low | Odd ratio  1.91 (CI 1.17-3.13) | Age, sex, race and ethnicity, educational level, service component, and lifetime history of the specified mental health  condition as reported in the baseline survey and other trauma exposures |
| Chin 2019 | Taiwan | Nurses | Cross-sectional | 32.7±7.4 | Women | 2,734 | 1- Work hours  2- burnout  3-Job stress  4-Job dissatisfaction | 1-Suicidal ideation | 1-Brief Psychiatric Symptom Rating  Scale | Moderate | Moderate | Low | Low | Relative risk  Work hours  40-60h  1.3 (CI 1.0-1.7)  >60h  0.9 (CI 0.8-1.1)  Burnout  1.4 (CI 1.2-1.6)  Job stress  1.2 (CI 1.1- 1.4)  Job dissatisfaction  1.1 (CI 1.0-1.3) | Adjusted (factors are unknown) |
| Chireh 2023 | Canada | working population | Cross-sectional | ≥15 | 40.1% women | 23,757 | 1-Long working hours  2-job stress  3-Job dissatisfaction | 1-Suicidal ideation | 1-Composite International Diagnostic Interview | Moderate | Moderate | Low | Low | Relative Risk  Long working hours  1.01 (CI 0.92–1.09)  0.95 (CI 0.84-1.08)  job stress  1.29 (CI 1.16–1.43)  1.30 (CI 1.11–1.52)  Job dissatisfaction  1.26 (CI 1.16–1.38)  1.23 (CI 1.09–1.39)  Men  Long working hours  0.96 (CI 0.86–1.07)  0.95 (CI 0.80–1.12)  job stress  1.29 (CI 1.13–1.48)  1.46 (CI 1.18–1.82)  Job dissatisfaction  1.27 (CI 1.13–1.42)  1.27 (CI 1.07–1.50)  Women  Long working hours  1.07 (CI 0.92–1.23)  0.99 (CI 0.82–1.19)  job stress  1.26 (CI 1.06–1.51)  1.13 (CI 0.90–1.42)  Job dissatisfaction  1.25 (CI 1.08–1.44)  1.20 (CI 1.01–1.44) | Multivariate |
| Choi 2018 | USA | Working population | Longitudinal | 34-69 | 50% women | 578 | 1- job control  2- job demands  3-Job strain  4-Job insecurity  5-working hours | 1-Suicidal ideation | 1- Mood and Anxiety  Symptoms Questionnaire | High | Low | Low | Low | Odd ratio  job control  2.94 (CI 1.03–8.35)  job demands  1.99 (CI 0.61–6.43)  Job strain  4.08 (CI 1.47–11.34)  Job insecurity  2.36 (CI 0.41–13.72)  working hours  ≥41h  2.60 (CI 0.91–7.37) | age, marital status, race, family suicide history, suicidal ideation at T1 |
| Dalglish 2015 | France | young adults | Cross-sectional | 30.8±3.3 | 62.3% women | 1,134 | 1-Job insecurity | 1-Suicidal ideation | 1-One item | Low | Low | Moderate | Low | Odd ratio  2.24 (CI 1.08–4.63) | age, sex, education, living with partner, insufficient social support, alcohol abuse in the last 12 months, major depression, parental depression |
| Dalum 2022 | Norway | veterinarians | Cross-sectional | ≥20 | 69.6% women | 2,596 | 1-Emotional demands | 1-Suicidal ideation | 1-Paykel’s five-item questionnaire | Moderate | Low | Low | Low | Odd ratio*  Emotional demands  1.02 (CI 0.97-1.07) | gender, age, civil status, negative life events,  SCL-5, reality weakness, use of alcohol to cope, main field of work |
| Dias 2022 | Brazil | medical students | Cross-sectional | 23.8±4.0 | 57.9% women | 209 | 1-Burnout | 1-Suicidal ideation  2-Suicide attempt | 1-Beck Scale for Suicide Ideation | Low | High | Low | Low | Odd ratio  Suicidal ideation  2.40 (CI 1.53-3.77)  Suicide attempt  1.49 (CI 0.77-2.89) | Unadjusted |
| Dyrbye 2008 | USA | medical students | Longitudinal | Unspecified | 48.2% women | 2248 | 1-Burnout | 1-Suicidal ideation | 1-three questions | High | High | Moderate | Low | Odd ratio  2.33 (CI 1.47–3.70) | Unadjusted |
| Ejdesgaard 2015 | Denmark | Soldiers | Unknown | ≥20 | 6.2% women | 1,264 | 1-High social support at work  2-Workload | 1-Suicidal ideation  2-Suicide attempts | 1-questionnaireby Centre for Suicide  Research | High | Moderate | Moderate | Low | Odd ratio**  Suicidal ideation  Social support at work  0.53 (CI 0.12-0.93)  Workload  1.63 (CI 1.25-2.00)  Suicide attempts  Workload  2.82 (CI 2.03-3.60) | Multivariate |
| Feskanich 2002 | USA | Nurses | Longitudinal | 36-61 | Women | 94,110 | 1-Job stress | 1-Suicide mortality | 1-Death certificates | Low | Moderate | Low | Low | Relative risk  1.9 (CI 0.8-4.7) | age, smoking status and quantity, alcohol intake, coffee consumption, marital status |
| Fresan 2019 | Mexico | Psychiatrists | Cross-sectional | 28-74 | 51% women | 288 | 1-Burnout | 1-Suicidal ideation | 1-Interview | Low | Moderate | Low | Low | Odd ratio  2.99 (CI 1.09-8.19) | Multivariate |
| Han 2021 | Korea | General population | Cross-sectional | ≥19 | Both % women not reported | 47,217 | 1-Long working hours  2-Shift work | 1-Suicidal ideation | 1-One item | Low | Low | Moderate | Low | Odd ratio  41–50h  1.15 (CI 0.82-1.63)  51-60h  1.48 (CI 0.99-2.21)  >60h  2.30 (CI 1.54-3.45)  Shift work  1.04 (CI 0.70-1.53) | age, sex and environmental factors: Smoking status, alcohol consumption, educational level, household  income, marital status, perceived health status, occupation, sleep duration, physical activity, comorbidities |
| Ishikawa 2022 (1) | Japan | resident physicians | Cross-sectional | ≥25 | 37.1% women | 4,306 | 1-Long working hours | 1-Suicidal ideation | 1-Items from survey | High | Moderate |  | Low | Odd ratio  40–59h  2.02 (CI 1.12-3.62)  60-79h  2.23 (CI 1.22 - 4.07)  80-99h  2.72 (CI 1.45 - 5.07)  >100h  3.64 (CI 1.88 -7.04) | Multiple |
| Ishikawa 2022 (2) | Japan | obstetrician/gynaecologists | Cross-sectional | Unspecified | 50% women | 919 | 1-Long working hours | 1-Suicidal ideation | 1-Japanese version (QIDS-SR-J) of the  16-item Quick Inventory of Depressive Symptomology–  Self-Report | High | Moderate | Low | Low | Odd ratio  60–79h  1.42 (CI 0.46–4.38)  80–99h  2.60 (CI 0.80–8.43)  ≧100h  7.03 (CI 1.95–25.38) | Multivariate |
| Jesus 2023 | Portugal | healthcare  professionals | Cross-sectional | 22-77 | 80.1% women | 1,172 | 1-Burnout | 1-Suicidal behaviour | 1-Suicidal Behaviours Questionnaire-Revised | Low | High | Low | Low | r  Disengagement  1172 (0.32)  Exhaustion  1172 (0.35) | Unadjusted |
| Kang 2017 | Korea | Workers | Cross-sectional | Unspecified | 48.3% women | 14,226 | 1-Shift work | 1-Suicidal ideation | 1-One item | Moderate | Moderate | Moderate | Low | Odd ratio  1.186 (CI 1.183-1.189)  Men  1.099 (CI 0.824 1.467)  Women  1.246 (CI 0.821 1.89) | age, gender, BMI, workplace, educational level, and marital status with or without young children under 6 years of age |
| Kim 2018 | Korea | Workers | Cross-sectional | 15-64 | 16.9% women | 53,969 | 1- Job demand  2- job control  3-Interpersonal conflict  4- Job insecurity  5- Lack of reward  6-Occupational climate | 1-Suicidal ideation | 1-One item | Low | Low | Moderate | Low | Odd ratio  Men  Job demand  1.00 (CI 0.91–1.10)  job control  1.27 (CI 1.03–1.57)  Interpersonal conflict  1.05 (CI 0.94–1.17)  Job insecurity  1.14 (CI 0.95–1.38)  Lack of reward  1.28 (CI 1.16–1.40)  Occupational climate  0.94 (CI 0.73–1.22)  Women  Job demand  0.95 (CI 0.83–1.10)  job control  0.99 (CI 0.73–1.32)  Interpersonal conflict  1.22 (CI 1.05–1.44)  Job insecurity  1.16 (CI 0.97–1.39)  Lack of reward  1.11 (CI 0.96–1.27)  Occupational climate  1.10 (CI 0.82–1.48) | age, marital, education, AUDIT, smoking, CESD |
| Kim 2017 | Korea | Workers | Longitudinal | 24-56 | 41.5% women | 2,912 | 1-job security | 1-Suicidal ideation | 1-One item | Low | Low | Moderate | Low | Hazard ratio***  Men  3.25 (CI 1.72 -6.16)  Women  1.55 (CI 0.93–2.59) | age, and score of CES-D at baseline as continuous variable, and marital status, education, annual household income, head of household, parental status, chronic disease, alcohol consumption, smoking, type of work, enterprise size, and full-time work as categorical variable |
| Kyron 2021 | Australia | police and emergency  services employees | Cross-sectional | Unspecified | Both % women not reported | 14,868 | 1-workplace bullying  2-Work/family conflict  3-Work–life balance | 1-Suicidal ideation  2-Suicide attempts | 1-Six questions | High | Moderate | Moderate | Low | Odd ratio  Suicidal ideation  workplace bullying  1.38 (CI 1.22- 1.57)  Work/family conflict  1.79 (CI 1.58- 2.02)  Suicide attempts  workplace bullying  1.68 (CI 1.06-2.56)  Work/family conflict  0.88 (CI 0.54-1.43) | Multivariate |
| Langhinrichsen-Rohling 2011 | USA | Air Force | Unknown | 31.78 ±7.38 | 20.7% women | 52,780 | 1-working hours | 1-Suicidal ideation | 2- two items | Low | Moderate | Moderate | Low | Odd ratio*  Men  1.11 (CI 1.03-1.18)  Women  1.11 (CI 1.03-1.18) | Multivariate |
| Leach 2020 | Australia | Workers | Cross-sectional | 52–58 | 51.7% women | 1,488 | 1-workplace bullying  2-Long working hours  3- job control  4- job demands  5- job insecurity | 1-Suicidal ideation | 1- Psychiatric Symptom  Frequency Scale | Low | Low | Low | Low | Odd ratio  workplace bullying  1.55 (CI 0.52–4.63)  Long working hours  0.90 (CI 0.38–2.12)  job control  1.20 (CI 0.64–2.25)  job demands  1.31 (CI 0.66–2.58)  job insecurity  2.51 (CI 1.40–4.50) | Socio-Demographic,  Health, and Current and  Prior Work Covariates, Excluding Those  with Prior  Suicidal Ideation |
| Li 2023 | China | medical residents | Cross-sectional | 24-27 | 68.1% women | 1,343 | 1-Long working hours | 1-Suicidal ideation | 1-Patient Health Questionnaire | Low | Low | Low | Low | Odd ratio  41-50h  0.82 (CI 0.48,1.40)  51-60h  0.98 (CI 0.57,1.68)  ≥61h  1.37 (CI 0.76,2.47) | Age, sex, and body mass index. sleep quality, have religions, experienced major events, history of chronic disease, visiting friend constantly and night shifts, alcohol habit |
| Lin 2020 | China | General population | Cross-sectional | 31.62± 7.43 | Women | 968 | 1-Long working hours  2-Job stress | 1-Suicidal ideation | 1-Patient Health Questionnaire | Low | Low | Low | Low | Odd ratio  Long working hours  1.260 (CI 0.805-1.973)  job stress  2.593 (CI 1.652-4.068) | age, education level, marital status, occupation type, physical exercise, alcohol consumption and smoking |
| Loerbroks 2016 | South Korea, China, Australia, and Germany | General population | Cross-sectional | 42.0 ± 10.2 | Both % women not reported | 12,422 | 1- job strain  2-job demand  3-job control | 1-Suicidal ideation | 1- Symptom Checklist-90  2-Zung Self-rating Depression  Scale  3-Patient Health  Questionnaire-9  4-Diagnostisches  Expertensystem für psychische Störungen  Stamm-Screening Questionnaire | Low | Moderate | Low | Low | Odd ratio  job strain  1.91 (CI 1.52-2.41)  job demand  1.79 (CI 1.43- 2.23)  job control  1.59 (CI 1.25-2.03) | age, gender, marital status,  and socioeconomic status |
| Lu 2022 | China | Nurses | Cross-sectional | 31.31±6.04 | 96.3% women | 1901 | 1-workplace bullying | 1-Suicidal ideation  2-Suicide attempts | 1- two items | Low | Moderate | Moderate | Low | Odd ratio  Suicidal ideation  3.19 (CI 2.41, 4.21)  Suicide attempts  5.25 (CI 3.69, 7.49) | Monthly income, somatic subhealth symptoms, sleep problems, anxiety, and depressive symptoms |
| Menon 2020 | USA | Physicians | Cross-sectional | Unspecified | 42.8% women | 1354 | 1- burnout | 1-Suicidal ideation | 1-One item | Moderate | Moderate | Moderate | Low | Odd ratio  0.88 (CI 0.64-1.22) | depression, sex, race/  ethnicity, training status, and age category |
| Milner 2017 (1) | Australia | General population | Case-Control | ≥15 | 26.3% women | 23,017 | 1- job demand  2-job control | 1-Suicide mortality | 1-International  Classification of Disease | Moderate | High | Low | Low | Odd ratio  Men  job demand  1.33 (CI 1.23-1.45)  job control  1.24 (CI 1.14-1.34)  Women  job demand  0.83 (CI 0.71-1.09)  job control  0.94 (CI 0.83 1.07) | Occupational  Skill Level |
| Milner 2016 | Australia | General population | Cross-Sectional | ≥18 | 44.9% women | 817 | 1- Social support at work  2-Quantitative demands  3-job control  4-Job insecurity | 1-Suicidal ideation | 1-Beck Depression Inventory | Moderate | Moderate | Low | Low | Odd ratio  social support  1.00 (CI 0.99–1.01)  job control**  1.01 (CI 0.99–1.02)  job demand  1.00 (CI 0.98–1.01)  Job insecurity  1.00 (CI 0.98–1.01) | Adjusted (factors are unknown) |
| Milner 2017 (2) | Australia | General population | Cross-Sectional | ≥18 | Men | 11,052 | 1-Job insecurity  2- job control  3- job demand | 1-Suicidal ideation | 1-Patient Health Questionnaire | Low | Moderate | Low | Low | Odd ratio  Job insecurity  1.69 (CI 1.44-1.99)  job control  1.15 (CI 1.05-1.26)  job demand  0.98 (CI 0.89-1.07) | Multivariable |
| Mo 2022 | China | migrant workers | Unknown | ≥18 | 63.2% women | 785 | 1-Workload  2-Role conflict  3-Job control  4- Social support at work  5-Working hours | 1-Suicidal ideation | 1-One item | Low | Low | Moderate | Low | Odd ratio*  Workload  1.06 (CI 1.02–1.11)  Role conflict  1.13 (CI 1.03–1.23)  Job control**  1.16 (CI 1.00–1.34)  Social support  1.01 (CI 0.78–1.30)  Working hours  1.00 (CI 0.97–1.02) | depression and the  sociodemographic variables |
| Nie 2022 | China | clinicians | Cross-Sectional | 25-60 | 54.8% women | 352 | 1-job stress | 1-Suicidal ideation | 1-Beck Depression Inventory | Low | High | Low | Low | r  352 (0.20) | Unadjusted |
| Niedhammer 2020 (1) | France | General population | Longitudinal | Unspecified | 46.6% women | 1,496,332 | 1-job strain  2-psychological demands | 2-suicide mortality | 1-death registry | Low | Moderate | Low | Low | Hazard ratio***  Men  job strain  1.28 (CI 1.09–1.51)  psychological demands  0.92 (CI 0.81–1.05)  women  job strain  1.35 (CI 1.03–1.77)  psychological demands  1.27 (CI 0.99–1.62) | calendar time, biomechanical, physical, chemical, and biological exposures |
| Niedhammer 2020 (2) | France | Employees | Cross-Sectional | 15-65 | 58% women | 20,430 | 1-job demands  2-job dissatisfaction  3-Work-family conflict  4-job insecurity  5-Long working hours  6-shift work | 1-Suicidal ideation | 1-one item | Low | Low | Moderate | Low | Odd ratio  Men  job demands  1.74 (CI 1.10- 2.76)  0.79 (CI 0.52- 1.22)  1.29 (CI 0.81- 2.07)  1.59 (CI 0.97- 2.58)  job dissatisfaction  1.71 (CI 1.06-2.76)  Work-family conflict  1.44 (CI 0.90- 2.31)  job insecurity  2.25 (CI 1.44-3.50)  Long working hours  0.76 (CI 0.42- 1.38)  shift work  1.28 (CI 0.62- 2.66)  Women  job demands  1.50 (CI 1.02- 2.20)  1.74 (CI 1.18- 2.59)  1.41 (CI 0.87- 2.28)  1.44 (CI 0.89-2.34)  job dissatisfaction  1.26 (CI 0.85-1.87)  Work-family conflict  1.08 (CI 0.76- 1.54)  job insecurity  1.17 (CI 0.80-1.71)  Long working hours  1.49 (CI 0.78- 2.88)  shift work  0.87 (CI 0.22- 3.41) | age, marital status, social support outside work, life events before the age of 18, life events within the last 3 years, occupation, and economic activity of the company |
| Nielsen 2016 | Norway | Employees | Longitudinal | 18-65 | 55% women | 1,939 | 1-workplace bullying | 1-Suicidal ideation | 1-Hopkins Symptoms  Checklist | High | Low | Moderate | Low | Odd ratio  6.41 (CI 1.85–22.14) | age, gender, baseline suicidal ideation and of the shared variance of the indicators of bullying behavior |
| Ostry 2007 | Canada | Workers | Longitudinal | Unspecified | Both % women not reported | 28,794 | 1-job control  2-Psychological demand  3-Physical demand  4- Social support at work | 1-suicide attempts  2-suicide mortality | 1-hospital discharge or  death record | Low | High | Low | Low | Odd ratio  suicide attempts  job control**  1.04 (CI 1.00-1.08)  Psychological demand  0.90 (CI 0.83-0.97)  Physical demand  0.89 (CI 0.66-1.21)  Social support  0.84 (CI 0.72-0.99)  suicide mortality  job control**  1.05 (CI 0.99-1.10)  Psychological demand  0.76 (CI 0.67-0.85)  Physical demand  1.04 (CI 0.73-1.50)  Social support  0.92 (CI 0.75-1.13) | Unadjusted |
| Park 2019 | Korea | Firefighters | Cross-Sectional | 42.51±9.10 | 7.4% women | 45,555 | 1-job stress | 1-Suicidal ideation | 1-Suicidal Behavior Questionnaire-Revised | Low | Moderate | Low | Low | Odd ratio  1.191 (CI 1.164–1.219) | Multivariable |
| Park 2022 | Korea | Workers | Cross-Sectional | Day worker  40.55 ± 6.71  Shift worker  37.40 ± 6.62 | 43.02% women | 79,009 | 1- Shift work | 1-Suicidal ideation | 1-one item | Low | Low | Moderate | Low | Odd ratio  0.942 (CI 0.858–1.034)  Men  1.157 (CI 1.021–1.310)  Women  0.957 (CI 0.826–1.109) | age, sex, education level, marital status, body mass index, Center for Epidemiologic Studies Depression Scale and Beck Anxiety Inventory |
| Park 2020 | Korea | Employees | Cross-Sectional | 20-35 | 51.1% women | 3,332 | 1-Long working Hours | 1-Suicidal ideation | 1-one item | Low | Moderate | Moderate | Low | Odd ratio  41–50h  1.98 (CI 0.95-4.10)  51-56h  3.48 (CI 1.48–8.19)  >60h  5.30 (CI 1.61–17.42) | gender, marriage status, residential area, educational level |
| Petrie 2020 | Australia | junior doctors | Cross-Sectional | ≥26 | 58.5% women | 2,706 | 1-Working Hours | 1-Suicidal ideation | 1-one item | High | Low | Moderate | Low | Odd ratio  45-49h  1.40 (CI 0.95 - 2.06)  50-54h  1.58 (CI 1.10 - 2.27)  >50h  2.00 (CI 1.42 - 2.81) | Age, gender, stage of training, location, area of specialty, marital status and whether medical degreewas completed overseas. |
| Petrie 2021 | Australia | junior doctors | Cross-Sectional | ≥26 | 61% women | 3,053 | 1-workplace bullying | 1-Suicidal ideation | 1-one item | High | Low | Moderate | Low | Odd ratio  2.92 (CI 1.42–6.03) | age, gender, location, stage of training, number of children living at home, whether training was undertaken overseas |
| Ross 2022 | Australia | industry apprentices | Cross-Sectional | Unspecified | 10.2% women | 1402 | 1-Workplace bullying | 1-Suicidal ideation | 1-one item | High | Moderate | Moderate | High | Odd ratio  1.17 (CI 0.79-1.75) | Multivariate |
| Son 2021 | Korea | Workers | Cross-Sectional | 22-40 | Women | 508 | 1-Shift work  2-Job stress | 1-Suicidal ideation | 1-one item | Low | Moderate | Moderate | Moderate | Odd ratio  Shift work  2.38 (CI 1.31–4.32)  Job stress  3.30 (CI 1.82–6.00) | age, smoking, alcohol, International Physical Activity Questionnaire |
| Stanley 2018 | USA | Firefighters | Unknown | 20-63 | 4.8% women | 831 | 1-job stress | 1-Suicidal ideation | 1-Suicidal Behaviors Questionnaire-Revised | Low | High | Low | Low | r  831 (0.265) | Unadjusted |
| Sterud 2008 | Norway | Ambulance personnel | Cross-Sectional | 18-66 | 23.2% women | 1,180 | 1-job dissatisfaction  2-Workplace bullying  3-burnout | 1-Suicidal ideation | 1-Paykel scale | High | Moderate | Low | Low | Odd ratio  job dissatisfaction  0.80 (CI 0.60-1.00)  Workplace bullying  1.60 (CI 1.00-2.70)  Burnout  1.50 (CI 1.10-2.00)  0.80 (CI 0.60-1.00)  1.30 (CI 1.00-1.70) | Adjusted (factors are unknown) |
| Sugawara 2013 | Japan | Workers | Cross-Sectional | 40-60 | 38.2% women | 5,878 | 1-job demand  2-job control  3-interpersonal conflict | 1-Suicidal ideation | 1-one item | High | Moderate | Moderate | Low | Odd ratio  Men  job demand  0.92 (CI 0.70-1.21)  job control  1.20 (CI 0.92-1.57)  Interpersonal conflict  1.15 (CI 0.88-1.51)  Women  job demand  1.22 (CI 0.87-1.72)  job control  1.19 (CI 0.86-1.65)  Interpersonal conflict  0.87 (CI 0.63-1.20) | Adjusted (factors are unknown) |
| Takada 2009 | Japan | business establishments | Cross-Sectional | 42.3 ±11.11 | Men | 2,834 | 1-job stress | 1-Suicidal ideation | 1-one item | High | High | Moderate | Low | Odd ratio  1.76 (CI 1.32–2.34)  2.16 (CI 1.64–2.85) | Age |
| Takeuchi 2014 | Japan | General population | Unknown | Unspecified | 52.9% women | 332,183 | 1-working hours | 1-Suicidal ideation | Unknown | Low | High | Moderate | Low | r  Men  156364 (0.587)  Women  175819 (0.134) | Age |
| Takusari 2011 | Japan | Workers | Cross-Sectional | ≥18 | 24.5% women | 3,233 | 1-Shift work | 1-Suicidal ideation | 1- Zung Self-rating Depression Scale | Low | Moderate | Low | Low | Odd ratio  Men  1.182 (CI 0.529–2.642)  Women  0.387 (CI 0.116–1.288) | Adjusted (factors are unknown) |
| Tsuno 2022 | Japan | Workers | Cross-Sectional | 45.7 ±13.8 | 41.4% women | 16,384 | 1-workplace bulling  2-physical demands  3-psychological demands  4-working hours | 1-Suicidal ideation | 1-one item | Low | Low | Moderate | Low | prevalence ratio*  workplace bulling  2.13 (CI 1.94 -2.34)  Men  2.17 (CI 1.92- 2.46)  Women  2.08 (CI 1.81- 2.40)  Men  physical demands  1.87 (CI 1.67 - 2.11)  psychological demands  2.24 (CI 2.00 - 2.51)  working hours  45-49h  0.76 (CI 0. 608 - 0.98)  50-59h  0.91 (CI 0.71 - 1.181)  ≥60h  0.99 (CI 0.77 - 1.27)  Women  physical demands  1.84 (CI 1.62 - 2.08)  psychological demands  2.18 (CI 1.92 - 2.47)  working hours  45-49h  1.07 (CI 0.82 - 1.39)  50-59h  0.99 (CI 0.69 - 1.41)  ≥60h  0.91 (CI 0.62 - 1.34) | gender, age, residential area and having a partner, education, household income and employment status, industry, office size and job type, workplace bullying and a prior history of depression |
| Tsutsumi 2007 | Japan | workers | Cohort | 41-72 | Men | 3,125 | 1-job control  2-job demand | 1-suicide mortality | 1-Cause-of-Death Register | Moderate | Low | Low | Low | Relative risk  job control  4.10 (CI 1.31–12.83)  job demand  0.73 (CI 0.22–2.38) | age, occupation, study area, marital status, education, smoking, alcohol consumption, and total cholesterol |
| Tyssen 2001 | Norway | Physicians | Longitudinal | 24–49 | 57% women | 522 | 1-job stress  2-Long working hours | 1-Suicidal ideation | 1-Paykel’s scale | High | Moderate | Low | Low | Odd ratio  job stress  1.03 (CI 1.00-1.06)  working hours  0.94 (CI 0.89-0.99) | Adjusted (factors are unknown) |
| Wall 2014 | Italy  Sweden | Surgeons | Cross-Sectional | Unspecified | 33.7% women | 421 | 1-role conflict  2-Work-family interface | 1-Suicidal ideation | 1-one item | High | High | Moderate | Low | Odd ratio  role conflict  1.85 (CI 1.17 – 2.91)  Work-family interface  1.59 (CI 1.03 – 2.48)  1.68 (CI 1.08 - 2.60) | Unadjusted |
| Wang 2016 | China | relief workers | Retrospective | 22-61 | 25.7% women | 65 | 1-working hours  2-work-family conflict  3- burnout | 1-Suicidal ideation | 1-one item | High | High | Moderate | Low | Odd ratio  working hours  0.317 (CI 0.106- 0.952)  work-family conflict  1.279 (CI 0.494-3.313)  burnout  0.238 (CI 0.040-1.417) | Unknown |
| Yoon 2016 | Korea | Workers | Cross-Sectional | Unspecified | 58.7% women | 1,995 | 1-job demand  2-job control | 1-Suicidal ideation | 1-one item | Low | Low | Moderate | Low | Odd ratio  Men  job demand  1.98 (CI 1.14-3.45)  job control  1.60 (CI 0.75-3.45)  Women  job demand  1.86 (CI 1.26-2.73)  job control  1.27 (CI 0.83-1.95) | age, house hold income, pain worker, smoking, alcohol drinking, physical activity |
| Younès 2018 | France | persons  attending primary care | Cross-Sectional | 18-65 | 53.6% women | 2,027 | 1-emotional demands  2-job insecurity | 1-death ideation, suicide ideation,  suicidal attempt | 1-Mini International Neuropsychiatric Interview | Low | Low | Low | Low | Odd ratio  Men  emotional demands  1.13 (CI 0.82 – 1.56)  job insecurity  0.88 (CI 0.61-1.28)  Women  emotional demands  1.35 (CI 1.01-1.80)  job insecurity  0.88 (CI 0.65 - 1.18) | age, occupational group, family status, level of education, past unemployment, present depression, alcohol use |

*Calculated by author(s) **Reversed by author(s) ***not include into meta-analysis
